# Two-band optical gain and ultrabright electroluminescence from colloidal quantum dots at 1000 A cm^−2^

**DOI:** 10.1038/s41467-022-31189-4

**Published:** 2022-06-29

**Authors:** Heeyoung Jung, Young-Shin Park, Namyoung Ahn, Jaehoon Lim, Igor Fedin, Clément Livache, Victor I. Klimov

**Affiliations:** 1 Nanotechnology and Advanced Spectroscopy Team, C-PCS, Chemistry Division, Los Alamos National Laboratory, Los Alamos, NM 87545 USA; 2 Centre for High Technology Materials, University of New Mexico, Albuquerque, NM 87131 USA; 3 Department of Energy Science and Centre for Artificial Atom, Sungkyunkwan University, Natural Sciences Campus, Seobu-ro 2066, Jangan-gu, Suwon, 16419 Gyeonggi-do Republic of Korea

**Keywords:** Nanoscale devices, Lasers, LEDs and light sources

## Abstract

Colloidal quantum dots (QDs) are attractive materials for the realization of solution-processable laser diodes. Primary challenges towards this objective are fast optical-gain relaxation due to nonradiative Auger recombination and poor stability of colloidal QD solids under high current densities required to obtain optical gain. Here we resolve these challenges and achieve broad-band optical gain spanning the band-edge (1S) and the higher-energy (1P) transitions. This demonstration is enabled by continuously graded QDs with strongly suppressed Auger recombination and a current-focusing device design, combined with short-pulse pumping. Using this approach, we achieve ultra-high current densities (~1000 A cm^−2^) and brightness (~10 million cd m^−2^), and demonstrate an unusual two-band electroluminescence regime for which the 1P band is more intense than the 1S feature. This implies the realization of extremely large QD occupancies of up to ~8 excitons per-dot, which corresponds to complete filling of the 1S and 1P electron shells.

## Abstract (web summary)

Nonradiative Auger recombination and poor stability of colloidal-quantum-dot solids at high current densities are primary obstacles to the realization of laser diodes based on these materials. Jung et al. achieve the current densities in excess of 1,000 A cm^−2^, which allows them to boost brightness to ~10 million cd m^−2^ and to produce strong, two-band optical gain.

## Introduction

Due to multi-fold degeneracy of QD band-edge states, realization of optical gain requires that at least a fraction of the QDs in an active medium are excited with multiexcitons^1–4^. In particular, if the band-edge states are two-fold degenerate, optical gain develops only if the average per-dot excitonic number, 〈*N*〉, is greater than 1, and the band-edge gain reaches saturation when all dots in a medium are populated with two or more excitons, that is, 〈*N*〉 ≥ 2. The reliance of optical gain on multiexcitons leads to a serious complication due to multicarrier Auger decay during which the energy of an excited electron-hole pair is quickly transferred nonradiatively to a third carrier residing in the same dot^5^. This limits optical gain lifetimes to very short timescales (typically, tens to hundreds of picoseconds) and greatly complicates realization of lasing with continuous-wave (cw) optical pumping^6^ or direct current (d.c.) electrical excitation^7^.

The problem of fast Auger decay can be alleviated using recently developed ‘continuously graded’ CdSe/Cd_*x*_Zn_1-*x*_Se QDs (cg-QDs), wherein Auger recombination is strongly suppressed due to elimination of sharp discontinuities in the confinement potential^7^. These dots have shown excellent performance as optical-gain materials and, in particular, allowed for the demonstration of band-edge (1S) optical gain with electrical excitation^7^ and the realization of dual-function devices that operate as both an optically pumped laser and a standard light-emitting diode (LED)^8^.

To elucidate current densities (*j*) required to enact electrically pumped lasing with cg-QDs, we use a model of ref. ^9^ which considers interplay between optical absorption and stimulated emission in a system of three QD states: an unexcited ground state, a single exciton, and a biexciton ($|0\rangle$, $|{{{{{\rm{X}}}}}}\rangle$, and $|{{{{{\rm{XX}}}}}}\rangle$, respectively). The corresponding probabilities (*P*_0_, *P*_X_, and *P*_XX_) are normalized so as *P*_0_ + *P*_X_ + *P*_XX_ = 1. When a QD is excited with a single exciton, the rate of the emitting $|{{{{{\rm{X}}}}}}\rangle$→$|0\rangle$ transition is identical to that of the iso-energetic absorbing $|{{{{{\rm{X}}}}}}\rangle$→$|{{{{{\rm{XX}}}}}}\rangle$ transition (here we neglect weak exciton-exciton interactions that do not appreciably shift this transition^10^). This implies that the single-exciton state is ‘gain neutral’ and, hence the magnitude of optical gain (*G*) is defined by the difference of the rate of stimulated emission due to the $|{{{{{\rm{XX}}}}}}\rangle \to |{{{{{\rm{X}}}}}}\rangle$ transition and the rate of absorption due to the $|0\rangle$→$|{{{{{\rm{X}}}}}}\rangle$ transition, that is, *G* = *G*_0_(*P*_XX_ – *P*_0_), where *G*_0_ is the maximal or ‘saturated’ gain realized when all QDs in a medium are excited with biexcitons (*P*_XX_ = 1). Based on this expression, the gain threshold (*G* = 0) corresponds to *P*_XX_ = *P*_0_, which can be realized, for example, if a QD medium is uniformly populated with single excitons, that is, *P*_XX_ = *P*_0_ = 0 and *P*_X_ = 1 or 〈*N*〉 = 1. In a more realistic situation of Poisson distribution of carrier populations, realized with short-pulse pumping, the gain threshold shifts to 〈*N*〉 = 1.15 (ref. ^4^).

To describe the regime of electrical pumping, we introduce per-dot excitation rate *g*, related to the current density by *g* = *σ*_e_(*j/e)*, ref. ^4^. Here *σ*_e_ is the electrical cross-section of a QD, which can be expressed via its geometrical cross-section (*σ*_g_ = *πR*^2^; *R* is the QD radius) and the areal QD filling factor (*f*) as *σ*_e_ = *f*^–1^*σ*_g_; ref. ^4^. In the case of steady-state excitation, probabilities *P*_0_, *P*_X_, and *P*_XX_ are connected to *g* and the exciton and biexciton lifetimes (*τ*_X_ and *τ*_XX_, respectively) by *P*_0_ = 1/*D*, *P*_X_ = $\widetilde{g}/D$, and ${P}_{{{{{{\rm{XX}}}}}}}={\widetilde{g}}^{2}/(\beta D)$, where $\widetilde{g}=g{\tau }_{{{{{{\rm{X}}}}}}}$, *β* = *τ*_X_/*τ*_XX_, and $D=1+\widetilde{g}+{\beta }^{-1}{\widetilde{g}}^{2}$ (ref. ^4^). Based on these expressions, the optical gain onset, presented in terms of the threshold current density (*j*_th,gain_), is ${j}_{{{{{{\rm{th}}}}}},{{{{{\rm{gain}}}}}}}={ef}/({\sigma }_{{{{{{\rm{g}}}}}}}\sqrt{{\tau }_{{{{{{\rm{X}}}}}}}{\tau }_{{{{{{\rm{XX}}}}}}}})$. We can also apply these expressions to estimate current density *j*_½_ required to achieve half-saturated optical gain (*G*=½*G*_0_), which yields ${j}_{1/2}={ef}\left(1+\sqrt{1+12{\tau }_{{{{{{\rm{XX}}}}}}}/{\tau }_{{{{{{\rm{X}}}}}}}}\right){(2{\sigma }_{{{{{{\rm{g}}}}}}}{\tau }_{{{{{{\rm{XX}}}}}}})}^{-1}$. Following ref. ^4^, we will use this quantity as an estimate of the lasing threshold.

For the purpose of illustration, we will apply the above expressions to standard medium-size, 3-nm radius CdSe QDs for which *τ*_X_ ≈ 20 ns (ref. ^11^), and *τ*_XX_ ≈ 110 ps (ref. ^5^). Using these parameters and assuming *f* = 0.5, we obtain *j*_th,gain_ = 190 A cm^−2^ and *j*_½_ = 2.6 kA cm^−2^. Both of these values are extremely high by standards of traditional QD LEDs that normally experience a breakdown at *j* of around 1−4 A cm^−2^ or less^12–15^.

Since a primary reason for high *j*_th,gain_ and *j*_½_ is very fast Auger decay, the use of cg-QDs with impeded Auger recombination should allow for reducing both of these quantities. Indeed, using parameters of cg-QDs from ref. ^7^ (*τ*_X_ ≈ 12 ns, *τ*_A,XX_ ≈ 2.4 ns, *σ*_g_ ≈ 3 × 10^–12^ cm^2^, and *f* = 0.5), we obtain *j*_th,gain_ ≈ 7 A cm^−2^ and *j*_½_ ≈ 27 A cm^−2^. As expected, these values are much lower than those for regular CdSe QDs, which is the result of both the suppression of Auger decay and the enhancement of the geometrical (hence, electrical) cross-section. The latter occurs due to a thick type-I shell, which helps capture injected carriers and funnel them into the small emitting core.

As we indicated earlier, the use of cg-QDs allowed for demonstrating electrically excited optical gain achieved in current-focusing LEDs at *j* > 3−4 A cm^−2^. Based on our estimations of *j*_½_, the realization of lasing would require considerably higher current densities (~30 A cm^−2^ and, possibly, more). A primary obstacle to reaching such values of *j* is device damage due to Joule heating caused by poor conductivity of solution-processed charge transport layers (CTLs) and a high contact resistance at various interfaces within a device stack. Device overheating can lead to degradation of QD emission efficiency due to thermally activated carrier trapping^16^, distortion of surface passivation, and generation of lattice defects, occurring especially readily at core/shell interfaces^15,16^. Other problems include thermal damage of an organic hole transport layer (HTL) due, for example, to a glass transition^17,18^, decline in material’s dielectric strength^19^, and delamination of metal contacts^20^.

Here, we demonstrate colloidal QD LEDs that are capable of generating current densities of more than 1,000 A cm^−2^ without irreversible device breakdown. As a result, we boost the device brightness to ~10 million cd m^−2^. The corresponding per-dot occupancy reaches ~8 excitons per dot, indicating complete population inversion of both the band-edge (1S) and the higher-energy (1P) transition. This unprecedented regime is achieved using cg-QDs incorporated into current-focusing devices driven by short electrical pulses. The graded potential of the cg-QDs leads to suppressed Auger decay, which simplifies the realization of high-order multiexciton states by reducing the required current densities. Concurrently, the use of current focusing and pulsed pumping helps limit heat build-up in the active QD volume, which improves device stability at high *j*.

## Results

### Realization of high current density LEDs

In principle, heat buildup in an operating device can be suppressed by spatially confining the injection area (*A*). This ‘current-focusing’ approach reduces the overall amount of heat-generating electrical power and simultaneously boosts heat exchange with the environment^21,22^. In particular, in a simple model of a uniformly heated active volume (Supplementary Note 1), the change in a device temperature during operation (*ΔT*) can be related to *A* and the heat exchange constant (*K*) by *ΔT* = *AjVK*^−1^, where *V* is the device bias^22^. Another approach to limit the amount of excess heat is by using pulsed excitation which can, in principle, further reduce overheating by a factor of around *τ*_p_/*τ*_T_, where *τ*_p_ is the pulse duration and *τ*_T_ = *C*/*K* is a characteristic heat dissipation time (*C* is the heat capacitance of the active device volume).

**Fig. 1:**
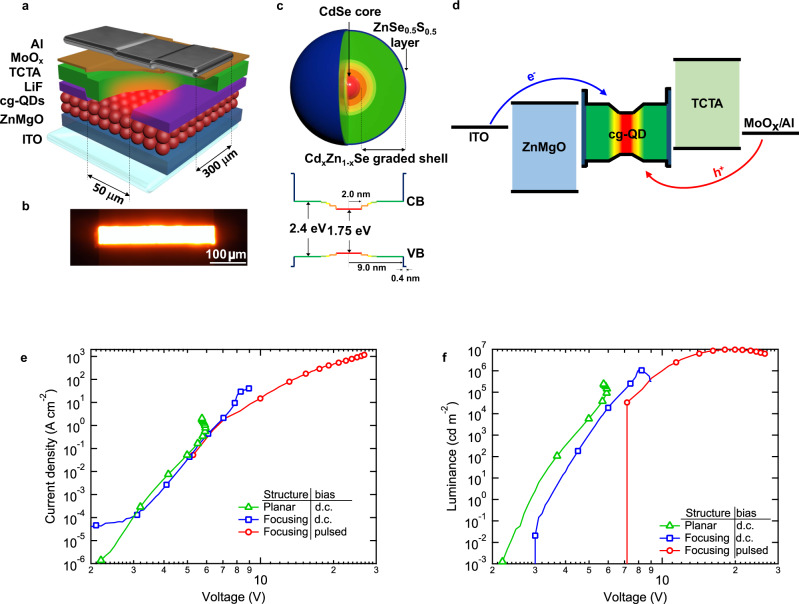
Ultrahigh current densities and brightnesses realized with current-focusing, pulsed cg-QD LEDs. **a** An LED device stack used in the present work. An emitting layer of cg-QDs is sandwiched between a ZnMgO ETL and a TCTA HTL. The HTL is separated from the emitting layer by a current-focusing aperture (50-μm-wide slit) in a LiF interlayer. To enhance the current focusing effect, the Al anode is prepared as a 300-μm-wide strip, oriented orthogonally to the slit. **b** An optical microscope image of a biased device (*V* = 5.0 V) shows that the emitting area is confined to 300×50 μm^2^ dimensions. **c** The internal structure of the cg-QDs and the corresponding conduction- and valence-band (CB and VB, respectively) confinement potentials. **d** A simplified energy band diagram of the cg-QD-based LED and a schematic illustration of electron (blue arrow) and hole (red arrow) injections. **e**, **f** *j*−*V* (panel **e**) and *L*−*V* (panel **f**) characteristics of ‘planar’ (green triangles) and ‘current-focusing’ (blue squares and red circles) LEDs containing two layers of cg-QDs under d.c. (green triangles and blue squares) and pulsed (red circles) excitation. In the pulsed regime (*τ*_p_ = 1 μs, *f*_p_ = 100 Hz), the current density reaches 1170 A cm^−2^. The corresponding peak brightness is 9.8 × 10^6 ^cd m^−2^.

In the present study, we investigate the effect of both current focusing and pulsed excitation, as well as their combination, on device overheating and the maximal accessible current densities. The structure of our devices is depicted in Fig. 1a, b. Their active layer is composed of 1 to 4 layers of CdSe/Cd_x_Zn_1-x_Se/ZnSe_0.5_S_0.5_ cg-QDs (Fig. 1c and Supplementary Fig. 1) whose parameters are similar to those of dots used previously in refs. ^7,23^ (Methods). In particular, they exhibit a high photoluminescence (PL) quantum yield of ~80% and a short single-exciton lifetime (*τ*_X_) of 13.4 ns (Supplementary Fig. 2a). This latter value is shorter than *τ*_X_ of standard CdSe QDs (*τ*_X_ ≈ 20 ns; ref. ^11^), which has been attributed to a modified ‘fine’ structure of band-edge excitonic states caused by asymmetric compression of the emitting core^23^. Further, due to a thick radially graded shell, these dots exhibit a long biexciton lifetime of 1.2 ns (Supplementary Fig. 2b) and a large electrical cross-section of ~5 × 10^−12^ cm^2^. As we discussed earlier, these characteristics benefit applications of cg-QD as optical-gain materials and, in particular, should allow for a considerable reduction of optical gain and lasing thresholds compared to standard, nonengineered QDs.

We embed 1 to 4 layers of cg-QDs into a p-i-n LED with a so-called ‘inverted’ architecture wherein the QD emitters are sandwiched between an inorganic electron transport layer (ETL) and an organic HTL (Fig. 1d). For electron injection, we use an indium tin oxide (ITO) cathode followed by a sol-gel ZnO ETL doped with Mg. The addition of Mg helps suppress oxygen vacancy-related defects (known QD exciton quenchers^24^) and simultaneously partially impede electron injection (due to lowered electron mobility^25^ and the increased injection barrier^26^) to balance it with slower hole injection.

For hole injection, we use an Al/MoO_x_ anode followed by a thermally evaporated HTL made of tris(4-carbazoyl-9-ylphenyl)amine) (TCTA). In standard ‘planar’ devices, the HTL is in direct contact with the QDs. In this case, the injection region is defined by the intersection of a bottom ITO electrode, prepared as a 1.5-mm-wide strip, and an orthogonal 1.5-mm-wide top Al electrode. In ‘current focusing’ structures, the HTL is separated from the QD layer by a thin LiF interlayer with a 50-μm-wide slit. To achieve stronger current focusing, the width of the Al contact is reduced to 300 μm. As a result, the injection region is confined to the 300-by-50 μm^2^ dimensions. This corresponds to *A* = 0.015 mm^2^, which is 150 times smaller than in the planar structure (see an optical microscope image of EL from the current-focusing device in Fig. 1b).

In Fig. 1e, we display *j*−*V* characteristics of a planar (green triangles) and a current focusing (blue squares) LED with an active region made of 2 layers of cg-QDs. For both devices, we observe a fast increase in the current density, which follows a power-law dependence (*j* $\propto$ *V*^*n*^) with a large exponent (*n* ≈ 10), a signature of charge transport in the ‘trap-filled limit’^27,28^. In the planar structure, *j* increases up to 3.4 A cm^−2^ at which point the device experiences a breakdown.

The current focusing structure is capable of sustaining higher current densities and fails only at *j*_max_ = 58 A cm^−2^, which is 17 times higher than for a planar device. The maximal value of *j* also exceeds that of ref. ^7^ by a factor of ~3, a direct consequence of stronger 2D confinement of the injection area versus 1D confinement used previously.

We are able to access even higher current densities by combining current focusing with pulsed excitation. In particular, using a sequence of rectangular pulses with *τ*_p_ = 1 μs and repetition rate *f*_p_ = 100 Hz, we can push *j* to unprecedented values of up to 1,170 A cm^−2^ (red circles in Fig. 1e). Interestingly, even at these ultra-high current densities the devices do not fail but only exhibit degradation of the EL intensity, typically referred to as a ‘droop effect’.

The realized extremely high current densities directly translate into extraordinary levels of device brightness (*L*). In a planar d.c. structure, the maximal pre-breakdown brightness (*L*_max_) is ~2.5 × 10^5^ cd m^−2^ (Fig. 1f, green triangles). It increases to approximately 1 million cd per m^2^ in a d.c. current focusing device (Fig. 1f, blue squares), and is pushed further to almost 10 million cd per m^2^ in the case of pulsed excitation (*L*_max_ = 9.8 × 10^6^ cd m^−2^; Fig. 1f, red circles). This brightness exceeds *L*_max_ of previously reported QD devices by a factor of around ~6 (ref. ^15^) and is higher than the peak brightness demonstrated for solution-processable LEDs based on other materials including organic molecules (*L*_max_ = 1.3 × 10^6^ cd m^−2^; ref. ^29^) and perovskites (*L*_max_ = 7.6 × 10^6^ cd m^−2^; ref. ^30^).

### Elucidation of the breakdown mechanism

**Fig. 2:**
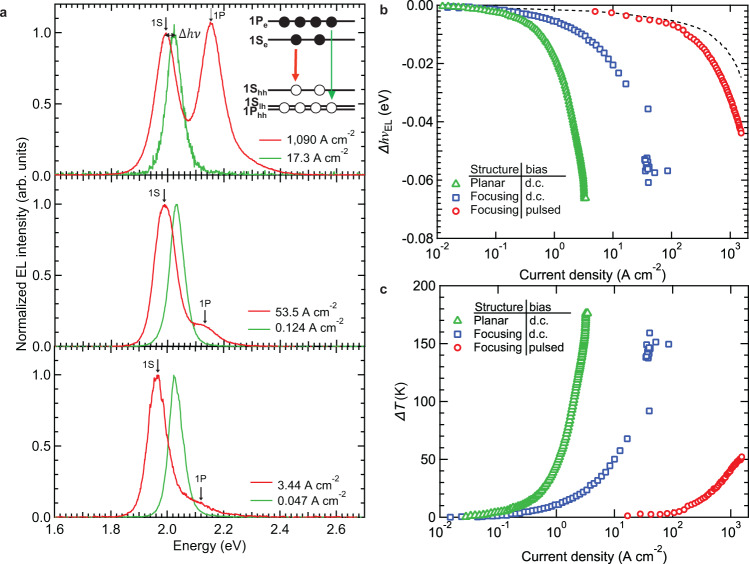
Effects of current focusing and d.c.-versus-pulsed excitation on EL spectra. **a** 1S-peak-normalized EL spectra at low (green) and high (red) current densities for the ‘planar’ LED (bottom, d.c. bias) versus the current-focusing device driven by a d.c. (middle) or a pulsed (top, *τ*_p_ = 1 μs and *f*_p_ = 100 Hz) bias. For all types of LEDs, the active region contains two cg-QD layers. The redshift of the band-edge 1S band (Δ*h*ν) is due to a combination of a Stark effect and Joule heating that prevail, respectively, at low and high *j*. Inset: Schematic illustration of optical transitions responsible for the band-edge (1S) and higher-energy (1P) EL features in the case of 6 injected excitons. Two of these excitons saturate the 1S band-edge transition and the remaining 4 are ‘forced’ into the higher-energy, sixfold degenerate 1P states. Subscripts hh and lh denote, respectively, heavy and light holes. The 1S_lh_ and 1P_hh_ levels are in close vicinity from each other; hence, higher-energy holes are able to sample both of these states. **b** The dependence of the shift of the 1S EL peak energy (*Δhv*_EL_) on *j* for the ‘planar’ (green triangles) and ‘current-focusing’ (blue squares and red circles) LEDs under d.c. (green triangles and blue squares) and pulsed (red circles) biases. The initial slow redshift of the EL peak due to a Stark effect (dashed line) is followed by a rapid decrease in the emission energy due to device heating. **c** Device overheating (*ΔT*) as a function of *j* derived from the EL data in panel **b** (same colour coding as in **b**).

In Fig. 2a, we display the measurements of EL spectra of the three types of devices. For a planar d.c. LED, at biases less than 5.4 V (*j* < 0.1 A cm^−2^), we observe single-band EL with a spectrum centered at *hv*_EL_ = 2.03 eV. This is similar to the spectral energy of the optically excited PL due to the band-edge transition, which couples the 1S_e_ electron and 1S_hh_ hole states (Fig. 2a, inset; subscripts ‘hh’ and ‘lh’ denote heavy and light holes, respectively). The increase in the bias leads initially to a gradual low-energy shift (*Δhv*_EL_) of the EL band (green triangles in Fig. 2b) characterized by an approximately quadratic dependence on applied electric field, a typical signature of a Stark effect (Fig. 2b, dashed line, and Supplementary Fig. 3)^31,32^. When bias exceeds 6.4 V (corresponding *j* is >0.4 A cm^−2^), the redshift accelerates and near breakdown (*V* = 11 V, *j* = 3.4 A cm^−2^), it becomes as large as ~66 meV. As observed previously^15^, the rapid change in the EL spectral energy at high *j* is due to reduction of the QD bandgap (*E*_g_) caused by the increase in the device temperature^15,33,34^.

The comparison of the PL spectra for 〈*N*〉 = 0.2 and 4.1 (Supplementary Fig. 4), measured with femtosecond laser pulses, does not show any discernable spectral shifts due to either thermal effects or multiexciton Coulomb interactions. This confirms that the redshift observed in the EL measurements in the regime of high current densities is primarily due to heating of the device active volume.

From our temperature-dependent PL measurements of cg-QDs (Supplementary Fig. 5), we obtain d*E*_g_/d*T* = 0.365 meV °C^−1^, which is consistent with previous measurements of traditional CdSe QDs^33,34^. Based on this value, at a near-breakdown voltage, the temperature of the active volume reaches 196 °C (Fig. 2c, green triangles), which corresponds to device overheating (*ΔT*) by 176 °C. While this is not expected to impart damage to a QD crystalline core^35^, it is sufficient to distort a surface passivating layer, leading to decline of the EL efficiency^16^. Furthermore, at *j* near *j*_max_, the temperature of the active volume becomes higher than that of the TCTA glass transition (*T*_g_ ≈ 151 C; ref. ^36^), due to which the device eventually fails. Previously, the damage of the organic HTL was cited as the main *j*-limiting factor for both QD-LEDs^15,37,38^ and organic LEDs^17,18^.

### Two-band electroluminescence and QD excitonic occupancies

In addition to a prominent redshift, another feature of high-*j* EL spectra is an additional higher energy band emerging at ~1 A cm^−2^ and growing to about 15% of the 1S band intensity at the highest *j* (Fig. 2a, bottom, and Supplementary Fig. 6). This feature is separated from the 1S emission by ~140 meV, which is consistent with the transition involving the excited 1P_e_ electron state (inset of Fig. 2a). Since the appearance of the 1P band requires complete saturation of the 2-fold-degenerate 1S_e_ level, this suggests that some of the dots in the active region contain 3 or more excitons. Indeed, according to our modeling of EL spectra (Supplementary Note 2), the fraction of such dots at a near-breakdown current density of ~3 A cm^−2^ is ~49%.

The EL spectra of the current-focusing device operating in a d.c. regime exhibit similar *j*-dependent trends (Fig. 2a, middle, and Fig. 2b, blue squares). In particular, they show the initial redshift of the band-edge EL due to a Stark effect, followed by a rapid reduction of *hv*_EL_ due to device overheating. However, as a result of current focusing, the onset of a sharp rise in the device temperature shifts to considerably higher *j* (~1.5 A cm^−2^) compared to planar structures, which is a direct consequence of the reduction of an injection area and simultaneous increase in a heat exchange constant. As *ΔT* ∝ *AK*^−1^, both of these factors contribute to reduced overheating (Fig. 2c, blue squares) and help boost *j*_max_ to nearly 50 A cm^−2^ (Fig. 2b, blue squares). This, in turn, leads to a higher relative intensity of the 1P band whose amplitude approaches ~31% of that of the 1S feature (Fig. 2a, middle, and Fig. 2b, blue squares). Based on our modeling (Supplementary Note 2), this corresponds to the fraction of dots with 3 and more exciton of ~79%, and 〈*N*〉 of ~3.6 (Supplementary Fig. 7). This value is higher than the 1S optical-gain threshold (〈*N*〉_th,gain_ ≈ 1.35, Supplementary Note 3 and Supplementary Fig. 7), indicating successful realization of population inversion of the band-edge transition.

In the case of a pulsed bias, the onset for overheating moves further to higher *j* (Fig. 2b, red circles). Specifically, for *τ*_p_ = 1 μs, a sharp decrease in *hv*_EL_, indicative of increasing *ΔT*, occurs only at current densities of 100 A cm^−2^. Importantly, due to the reduced heat buildup during the voltage pulse and nearly complete heat dissipation between the pulses in the 100 Hz pulse sequence (Supplementary Fig. 8), the overheating of the active area is limited to ~70 °C (Fig. 2c, red circles) even at the highest *j*. This implies that the device temperature always remains below *T*_g_ of the organic HTL. As a result, we are able to reach ultra-high current densities of more than 1,000 A cm^−2^ without causing device breakdown.

Due to extremely high *j*, we can realize an unusual EL regime when the higher-energy 1P feature becomes more intense than the band-edge 1S band (Figs. 2a, top, and 3a). This indicates that we able to achieve extremely high QD excitonic occupancies for which the band-edge 1S_e,h_ levels are completely saturated with two excitons, and the remaining carriers are pushed into the higher-energy 1P_e,h_ states. Importantly, such a peculiar two-band EL regime is not accessible with standard (nongraded) core/shell QDs (Supplementary Fig. 9), as fast (non-impeded) Auger decay limits the QD occupancy to ~2 excitons or less.

**Fig. 3:**
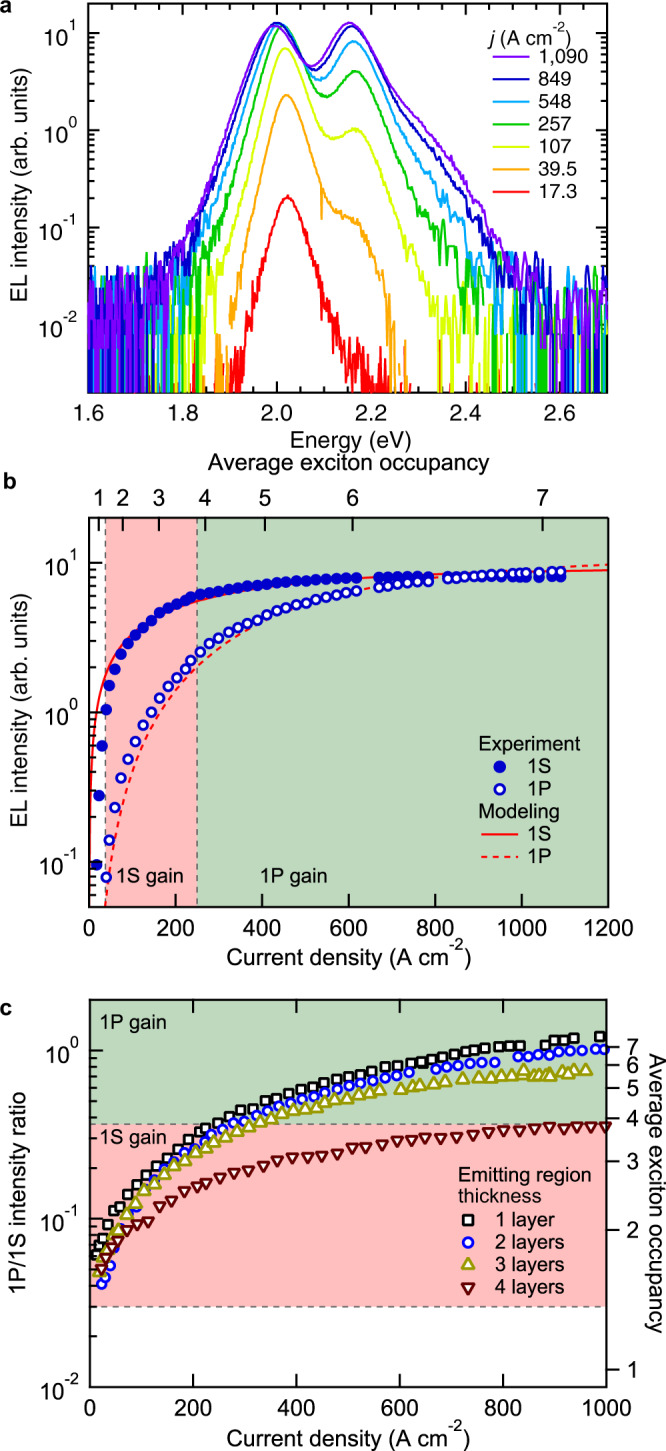
Quantification of per-dot excitonic occupancies based on two-band EL spectra. **a** The EL spectra of a current-focusing device under pulsed bias (*τ*_p_ = 1 μs, *f*_p_ = 100 Hz) as a function of *j*. **b** The 1S and 1P EL intensities (solid and open circles, respectively; determined from integrated areas) as a function of *j* inferred from the EL spectra in **a**. The lines are the calculations conducted using the ‘correlated-injection’ model of ref. ^7^ (Supplementary Note 2) with *β* = 1.37 × 10^7 ^C^−1^ cm^2^ and *τ*_XX_ = 1.5 ns (see Supplementary Fig. 15 for calculations conducted for *τ*_XX_ = 1.2 ns and 1.9 ns). The ranges of *j* that correspond to 1 S and 1 P optical gain (pink and green shadings, respectively) are determined based on the condition of achieving population inversion for the 1S and 1P transitions (〈*N*_th,gain_ = 1.35 and 3.84, respectively; see Supplementary Note 3). **c** The ratio of the 1P- and 1S-band EL intensities as a function of *j* for current focusing LEDs whose emissive layer contains 1 to 4 cg-QD layers. All devices are operated using pulsed bias with *τ*_p_ = 1 μs and *f*_p_ = 100 Hz. At *j* of ~1000 A cm^−2^, the 1P-to-1S intensity ratio is 1.24, 1.07, 0.75 and 0.37 for devices with the number of the cg-QD layers (*m*) increasing from 1 to 4. These values correspond to 〈*N*〉 of 7.8, 7.1, 5.7 and 3.9. In the case of the ideal, 100% EL quantum yield, 〈*N*〉 should change as 1/*m*. As a result of nonideal EL performance, the observed drop in 〈*N*〉 is slower than that predicated by the 1/*m* dependence.

In Fig. 3b, we model the measured 1 S and 1 P EL intensities (solid and open circles, respectively) within the ‘correlated injection’ framework of ref. ^7^ which takes into consideration Fermi distribution of carriers across QD states and radiative and nonradiative (Auger) rates derived based on experimentally measured PL dynamics (Supplementary Note 2, Supplementary Fig. 2, and Supplementary Table 1). The calculated 1S and 1P signals (Fig. 3b, solid and dashed lines, respectively) accurately describe the measurements and indicate that at the highest *j*, the per-dot excitonic occupancy reaches 7.1, which corresponds to complete saturation of the 2-fold degenerate 1S_e_ electron level and near complete filling of the 6-fold-degenerate 1P_e_ state.

### The effect of the QD layer thickness

In addition to devices with two cg-QD layers, we have also fabricated and analyzed LEDs that contain 1, 3, and 4 cg-QD layers (Fig. 3c and Supplementary Fig. 10). For all of them, we are able to reach current densities of ~1000 A cm^−2^, for which we observe the intense 1P band (Supplementary Fig. 11). Based on the measured 1P-to-1S intensity ratio, 〈*N*〉 reaches 7.8, 7.1, 5.7, and 3.9 for devices with 1 to 4 cg-QD layers (Fig. 3c and Supplementary Fig. 7). All of these values correspond to complete population inversion of the 1S transition, which is achieved when 〈*N*〉 is ca. 2 or more.

Nominally, the population inversion threshold for the 1P_e_−1P_h_ transition occurs when 〈*N*〉 is ~5, that is, when the 1P shell is half occupied. However, as was observed in ref. ^7^, the 1P gain threshold corresponds to the onset of population inversion for a weakly allowed 1P_e_−1S_hh_ transition. According to our calculations (Supplementary Note 3), for our cg-QDs, it occurs when 〈*N*〉 is ~3.8. This suggests, that even in the 4-layer device, in addition to inverting the 1S transition, we also achieve an optical gain threshold for the 1P transition (Fig. 3c). These results have important practical implications as a thicker QD layer is capable to deliver higher modal gain, which should simplify practical realization of the lasing effect.

### Tests of stability and reproducibility of device characteristics

Finally, we assess the stability of our ultra-high-*j* devices which is essential for realizing stable lasing. The repeating sweeps of an applied voltage between 2 and 40 V in forward and reverse directions do not show any noticeable hysteresis for either *j* or the EL intensity (Supplementary Fig. 12a, b). Further, our devices exhibit fairly good long-term stability. In particular, the LED driven using *j* of ~700 A cm^−2^ showed only an 18% drop in the current density after 10 h of operation. The device also preserved ~63% of its initial EL intensity at the end of the test (Supplementary Fig. 12c,d; *τ*_p_ = 1 μs, *f*_p_ = 100 Hz).

To evaluate the reproducibility of the device characteristics, we have fabricated and tested 8 LEDs containing 2 QD layers (Supplementary Fig. 13). With seven of the tested devices, we were able to reach current densities of greater than 1000 A cm^−2^ before breakdown. The observed maximal 1P-to-1S EL intensity ratio varied between 0.75 and 1.35 and on average was ~1. This implies that the maximal QD occupancy was ~6.8 excitons per dot on average.

### Optical-loss analysis

Modeling of the optical field distribution in the 4-QD-layer device indicates that the mode confinement factor for the active QD region (*Γ*_QD_) is ~23% (see Supplementary Table 2). Based on variable stripe length measurements, saturated 1S gain of cg-QDs can be as high as ~800 cm^−^^1^ (Supplementary Fig. 14a). This yields the modal gain of ~180 cm^−1^ for our devices. Optical losses in our structures are primarily due to light absorption in the bottom ITO layer. The corresponding mode confinement factor (*Γ*_ITO_) is ~56% which yields modal losses of around 250−300 cm^−^^1^ for standard ITO. However, the losses can be reduced approximately threefold (to *ca*. 80−100 cm^−^^1^; Supplementary Fig. 14b) with low-index ITO (L-ITO) applied in ref. ^8^. The use of L-ITO would further simplify achieving lasing due to the shift of the optical field profile from the ITO layer towards the QD region^8^. Thus, optical gain in the 4-QD-layer device can, in principle, outcompete optical losses in the bottom electrode by ~100 cm^−^^1^. Indeed, as was shown in ref. ^8^, even a 3-QD-layer active region yields modal gain which is large enough for achieving optically excited lasing in LED-like devices with an integrated distributed feedback (DFB) resonator.

## Discussion

The reported results represent a qualitative advance compared to the previous demonstration of electrically excited optical gain of ref. ^7^. The maximal current densities realized in that work (~18 A cm^−^^2^) allowed for inverting the population of the band-edge transition. However, they were not sufficient to reach the lasing regime. Further, in the previously reported devices, the active region was only one QD layer thick. As a result, it was not capable of supporting a waveguiding regime and, further, provided very low modal gain. These problems have been resolved in the present study.

The realized current densities (>1000 A cm^−^^2^) allow us to inject up to ~8 excitons per dot and thereby realize strong optical gain for both the band-edge (1S) and the excited-state (1P) transitions. Importantly, optical gain is maintained in devices with an active region of up to 4 QD layers capable of supporting a low-loss waveguided mode with a large (>20%) mode confinement factor^8^ and correspondingly large modal gain. The next step is to combine insights of the present study with those of ref. ^8^, which demonstrated a practical approach for incorporating an optical DFB cavity into a functional EL device. This represents a viable path towards a long-pursued objective – the realization of solution processable laser diodes.

## Methods

### Materials

Cadmium oxide (CdO, 99.99%), selenium (Se, −200 mesh, 99.999%), zinc acetate (Zn(ac)_2_, 99.99%), sulfur (S, 99%), oleic acid (OA, 90%), 1-octadecene (ODE, 90%), tri-n-octylphosphine (TOP, 97%), magnesium acetate tetrahydrate ((CH_3_COO)_2_Mg·4 H_2_O, 99.997%) and 2-methoxyethanol (CH_3_OCH_3_CH_3_OH, 99.3%) were purchased from Alfa Aesar. Ethanolamine (99.5%), Poly(amidoamine)dendrimers (PAD), methanol (99.8%) and toluene (99.8%) were purchased from Sigma Aldrich. Tris(4-carbazoyl-9-ylphenyl)amine (TCTA, 99.5%) was purchased from Lumtec. All chemicals were used as is without additional purification.

### Fabrication and characterization of continuously graded CdSe/Cd_*x*_Zn_1−*x*_Se/ZnSe_0.5_S_0.5_ quantum dots (cg-QDs)

0.5 M cadmium oleate [Cd(OA)_2_] and 0.5 M zinc oleate [Zn(OA)_2_] stock solutions were prepared by reacting 20 mmol of CdO or Zn(ac)_2_ with 20 ml oleic acid (OA) and 20 ml 1-octadecene (ODE) at 130 °C under vacuum. Trioctylphosphine selenium (2 M; TOPSe) and sulfur (2 M; TOPS) solutions were prepared by reacting 10 mmol of, respectively, Se and S with 5 ml of TOP at room temperature overnight. Cd(OA)_2_ and Zn(OA)_2_ were kept at 100 °C to prevent solidification.

The CdSe cores with a 2-nm mean radius were prepared using a modified literature procedure^39^. To grow graded Cd_*x*_Zn_1−*x*_Se shells around the cores, 0.27 μmol of fabricated CdSe particles were dispersed in 4 ml ODE and 1 ml TOP. After quickly increasing the temperature to 310 °C, 2 ml Zn(OA)_2_ was added to the reaction mixture and a precursor solution consisting of 4.5 ml Cd(OA)_2_, 4.5 ml TOPSe and 9 ml ODE was continuously fed into the reaction at 310 °C at a rate of 3 ml h^−1^. To prevent solidification of the precursors, 2 ml Zn(OA)_2_ was separately added into the reactor every 40 min. The overall reaction time required to synthesize CdSe/Cd_*x*_Zn_1−*x*_Se cg-QDs with the 9 nm radius reported in this study was 360 min. To overcoat these particles with a thin ZnSe_0.5_S_0.5_ shell, we added 6 ml Zn(OA)_2_, 0.75 ml TOPS and 0.75 ml TOPSe to the reaction mixture, and continued the growth for 20 min at 310 °C. The reaction was terminated by rapid cooling to room temperature. The fabricated cg-QDs were purified five times by a precipitation/redispersion procedure (ethanol/hexane) and finally dispersed in toluene at a concentration of 20 mg ml^−1^. All procedures were conducted under nitrogen atmosphere using a Schlenk-line approach.

The size and morphology of the synthesized cg-QDs were investigated using a JEOL 2010F high-resolution transmission electron microscope (TEM). The chemical composition of the QDs was studied using a Shimadzu ICPE-9000 system for inductively coupled plasma-atomic emission spectroscopy (ICP-AES). Absorption spectra of the QDs were acquired with an Agilent 8453 UV–Visible spectrometer. PL spectra and PL decay dynamics were measured using a Horiba Fluoromax-4 spectrofluorometer. Photoluminescence (PL) quantum yields (QYs) of the QDs were obtained by comparing their emission intensity with that of a reference rhodamine 101 dye (QY = 99% in ethanol).

### Fabrication and characterization of LEDs

A ZnMgO electron transport layer (ETL) was prepared using a sol-gel process. A sol-gel precursor was prepared by dissolving 916 mg of zinc acetate dihydrate (Zn(CH_3_COO)_2_·2H_2_O) and 81.4 mg of magnesium acetate tetrahydrate ((CH_3_COO)_2_Mg·4 H_2_O) in 10 mL of 2-methoxyethanol (CH_3_OCH_3_CH_3_OH). Then, 0.28 g of ethanolamine was slowly added to the stirred solution. The stirring of the resulting mixture continued overnight.

To fabricate an ETL, a glass substrate pre-patterned with indium tin oxide (ITO) electrodes was cleaned by treating it sequentially with acetone, isopropyl alcohol, and deionized water under sonication for 10 min. Afterwards, the substrate was dried with an N_2_ gas in an oven at 120 °C. A ZnMgO precursor solution was spun onto the ITO-patterned substrate side at 3000 rpm and annealed at 200 °C in ambient air for 2 hours.

Poly(amidoamine)dendrimers (PAD) ligands were used to define the number of cg-QD layers in an LED emitting region. A PAD ligand layer was prepared on top of a ZnMgO layer by spin-coating from a methanol solution (2 mg ml^−1^) at 4000 rpm, which was followed by washing with methanol to remove weakly bound ligand molecules. A solution of cg-QDs in toluene (20 mg ml^−1^) was deposited at 4000 rpm onto the ITO/ZnMgO/PAD substrate, which was then washed with toluene. These steps were repeated up to 4 times to prepare devices with an emitting region containing 1 to 4 cg-QD layers.

In ‘current-focusing’ devices, a 40-nm thick LiF spacer with a 50-μm gap was thermally evaporated onto the emitting layer using a metal shadow mask. The LiF deposition step was skipped during the fabrication of ‘planar’ LEDs. A 60-nm hole transport layer (HTL) made of tris(4-carbazoyl-9-ylphenyl)amine (TCTA) HTL was deposited by thermal evaporation under a vapor pressure of 10^−^^6^ torr at a rate of 0.4 Å s^−^^1^. The device was completed by depositing 10 nm of MoO_x_ followed by 100 nm of Al. Both layers were prepared via thermal evaporation (10^−^^6^ torr vapor pressure) at deposition rates of 0.1 and 1–2 Å s^−^^1^, respectively. The TCTA and MoO_x_ layers were fabricated without a shadow mask. The Al electrode was prepared using a shadow mask as a strip having a width of either 1.5 mm (‘planar’ devices) or 300 μm (‘current focusing’ devices).

In the case of direct current (d.c.) excitation, device characteristics were measured with a source measurement unit (Keithley 237). For a pulsed-excitation regime, a voltage pulse generated by an arbitrary-function generator (Tektronix, AFG320) was amplified by a highspeed bipolar amplifier (NF Corporation, HSA4101). The amplifier output was monitored with an oscilloscope (Tektronix, TDS 2004B) using a 1/100 voltage divider and a 50 Ω load resistor.

In both d.c. and pulsed electrical measurements, a cathode probe was connected to the center of a top ITO pad and an anode probe was connected to an Al pad. To obtain a voltage across the device (*V*), we subtracted voltage drops across the ITO line (from the cathode contact point to the device area) and the 50 Ω resistor from the applied voltage (Supplementary Fig. 16).

Electroluminescence (EL) spectra were measured using an Ocean Optics spectrometer (USB2000). The LED brightness (*L*) was inferred from photocurrent measurements conducted with a calibrated Si photodiode (Newport 818-UV and PDA10A2 for d.c. and pulsed measurements, respectively) using the following equation:

$$L={K}_{m}{I}_{{ph}}\left(\frac{{R}^{2}}{{A}_{{EL}}{A}_{{Si}}}\right)\frac{\int \left[{S}_{{EL}}\left(\lambda \right)/{S}_{{Si}}\left(\lambda \right)\right]V\left(\lambda \right)d\lambda }{\int {S}_{{EL}}\left(\lambda \right)d\lambda }$$

where *V*(λ) is the photopic response function, *K*_m_ is the corresponding scaling factor (683 lm W^−^^1^ at 555 nm), *I*_ph_, is a photocurrent of the Si photodiode, *A*_Si_ is its area, *S*_Si_(λ) is its responsivity, *R* is distance between the EL area of the tested LED and the Si photodiode, *A*_EL_ is the emitting area of the cg-QD LED, and *S*_EL_(λ) is its EL spectrum. The cg-QD LED emitting area was measured using an optical microscope (Fig. 1b).

### Measurements of temperature-dependent photoluminescence

A glass/ZnMgO substrate was prepared as described in the previous section. Then, 2 layers of cg-QDs were spin cast onto the substrate from a 20 mg ml^−^^1^ toluene solution. The sample was mounted onto a cold finger of a liquid-nitrogen cryostat with a thermal grease and kept under vacuum for the duration of the experiment. The sample temperature was controlled with a Lakeshore 335 instrument using the built-in 25 Ω heater and a temperature sensor mounted in the vicinity of the cg-QD film. The sample PL was excited by ~190 fs pulses derived from a regeneratively-amplified ytterbium-doped potassium gadolinium tungastate (Yb:KGW) laser (Pharos, Light Conversion). The fundamental 1030 nm output was tripled in a high-harmonic generator (HIRO, Light Conversion) to obtain the 343 nm pump pulses at 200 kHz. A 10 cm CaF_2_ lens was used to focus the beam onto the sample down to a 60 μm diameter spot. The per-pulse fluence was set to ~1 μJ cm^−^^2^. A time-integrated PL signal was collected by a lens in the direction normal to the cg-QD film and analyzed with a spectrograph (Acton SpectraPro 300i) coupled to a liquid-nitrogen-cooled charge coupled device camera (Roper Scientific LN-CCD-1340/400).

## Supplementary information

Supplementary Information

## Data Availability

The data that support the findings of this study are available from the authors on reasonable request.
