# Supplementary material for: Two-band optical gain and ultrabright electroluminescence from colloidal quantum dots at 1000 A cm−2
Source: Nat Commun. 2022 Jun 29;13:3734. doi: 10.1038/s41467-022-31189-4 (PMC9243032; doi:10.1038/s41467-022-31189-4)
Supplement: Supplementary file 1 — Supplementary Information [file 41467_2022_31189_MOESM1_ESM.pdf]

Supplementary Information

Two-Band Optical Gain and Ultrabright Electroluminescence from Colloidal Quantum Dots at 1000 A cm^{-2}

Heeyoung Jung[1], Young-Shin Park[1, 2], Namyoung Ahn[1], Jaehoon Lim[1,3], Igor Fedin[1], Clément Livache[1], and Victor I. Klimov[1]*

[1] Nanotechnology and Advanced Spectroscopy Team, C-PCS, Chemistry Division, Los Alamos National Laboratory, Los Alamos, New Mexico 87545, United States

[2] Centre for High Technology Materials, University of New Mexico, Albuquerque, New Mexico 87131, USA

[3]Department of Energy Science and Centre for Artificial Atom, Sungkyunkwan University, Natural Sciences Campus, Seobu-ro 2066, Jangan-gu, Suwon 16419, Gyeonggi-do, Republic of Korea

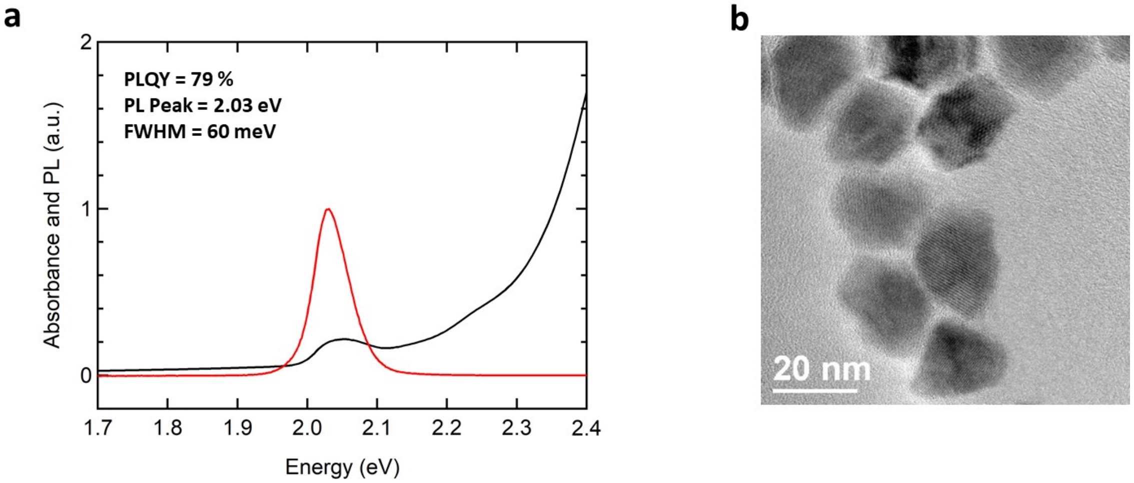

Supplementary Figure 1. Characterization of cg-QDs. a, Photoluminescence (PL) (red line) and linear absorption (black line) spectra of the cg-QDs prepared as a toluene solution. **b,** An exemplary transmission electron microscopy image of the cg-QDs.

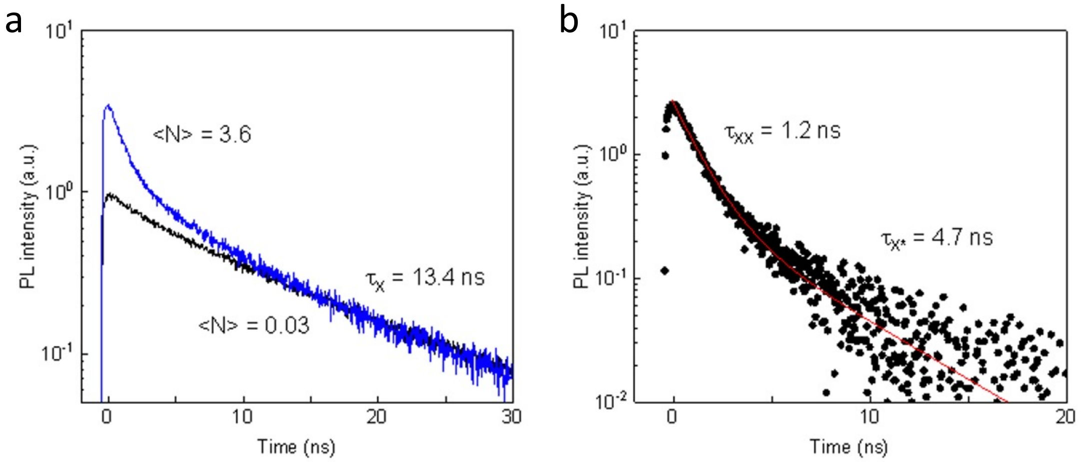

Supplementary Figure 2. PL dynamics of the cg-QDs. a, PL dynamics of the stirred cg-QD solution sample measured at the excitation level which correspond to the average per-dot excitonic occupancies $\langle N \rangle = 0.03$ (black line) and 3.6 (blue line). The sample is excited using 100 fs, 400-nm pulses with the pulse repetition rate of 250 kHz. The two traces are 'tail normalized' to match long-time (>30 ns) decays. Based on these measurements, the single-exciton lifetime (τ_X) is 13.4 ns, which yields $\gamma_{X,r} = 1/\tau_X = 0.075$ ns^{-1}. **b,** The dynamics obtained by subtracting the two traces in panel **a** (symbols) yield information on biexciton recombination (initial fast decay with the time constant τ_{XX}) and charged exciton recombination (slower, follow-up decay; τ_{X*}). Based on the double-exponential fit (red line), $\tau_{XX} = 1.2$ ns and $\tau_{X*} = 4.7$ ns.

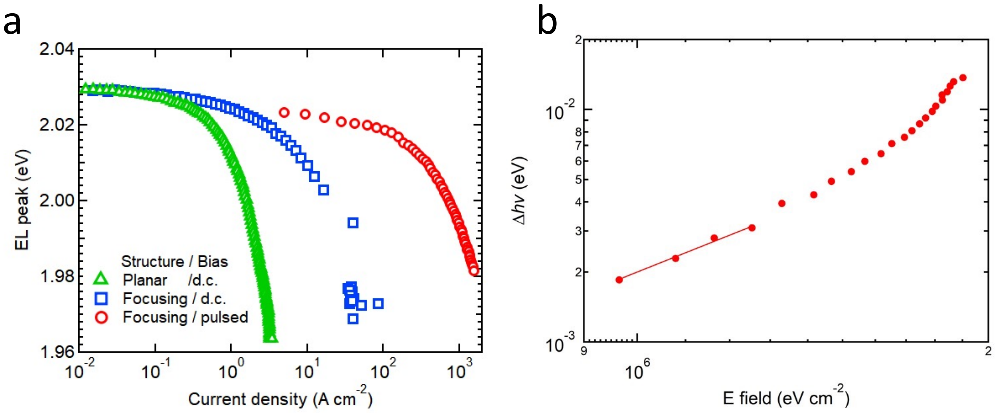

Supplementary Figure 3. The analysis of the initial 'slow' bias-dependent red shift of the EL spectra. a, The dependence of the 1S EL peak energy on j for the 'planar' (green triangles) and 'current-focusing' (blue squares and red circles) LEDs under d.c. (green triangles and blue squares) and pulsed (red circles) biases. **b,** The bias induced shift of the band-edge EL feature (symbols) is plotted as a function of electric field (F) using a log-log representation. These data were collected for a current-focusing device operating under pulsed excitation with the pulse duration $\tau_p = 1$ μs (Fig. 2, of the main article). At low F, the log-log slope of the observed dependence is 2 (line) which is a signature of the 'quadratic' Stark effect[1,2]. Based on these data, the polarizability of the cg-QDs is 2.0×10^{-6} meV cm^2 kV^{-2}.

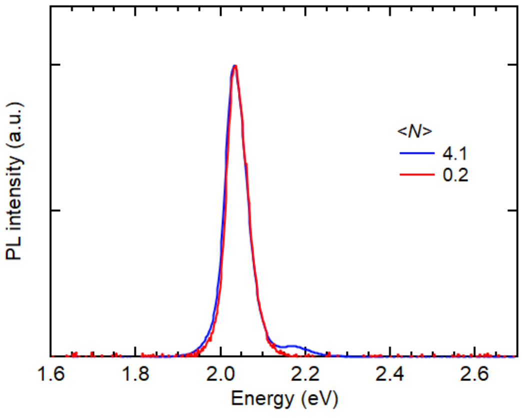

Supplementary Figure 4. Pump-intensity dependence photoluminescence spectra of cg-QDs. PL spectra of cg-QDs in solution under fs-pulse excitation with pump fluences that correspond to $\langle N \rangle = 4.1$ (blue) and $\langle N \rangle = 0.2$ (red). These spectra were collected using 400-nm excitation from a frequency-doubled, amplified femtosecond laser. The pulse-to-pulse interval was 100 μs to ensure complete relaxation of the QD medium between excitation events.

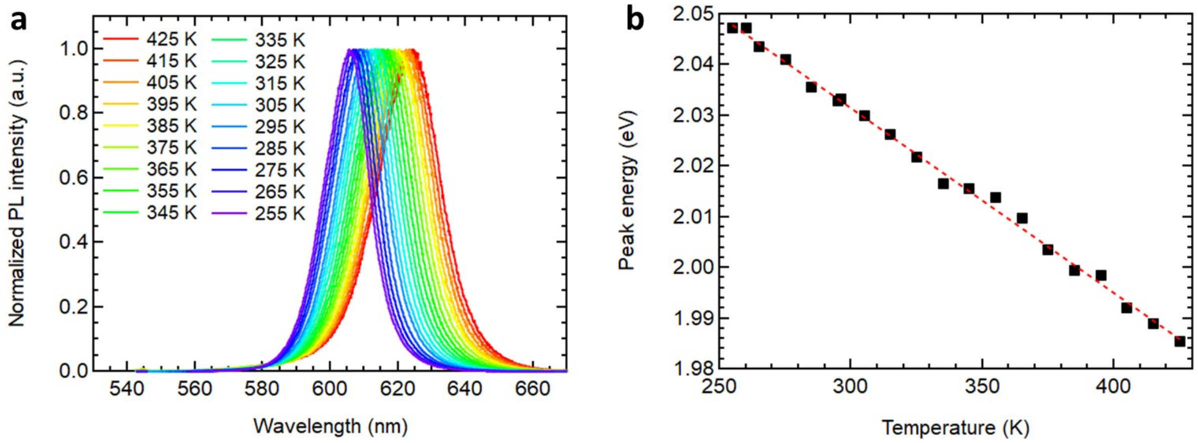

Supplementary Figure 5. Elucidation of the temperature dependence of the cg-QD bandgap. **a,** Temperature-dependent PL spectra of a cg-QD film. The sample is prepared as a close-packed film deposited onto a ZnMgO/glass substrate. **b,** The measured PL peak energy as a function of temperature, T (squares). Based on the linear fit (dashed line), $dE_g/dT = 0.365$ meV K^{-1}.

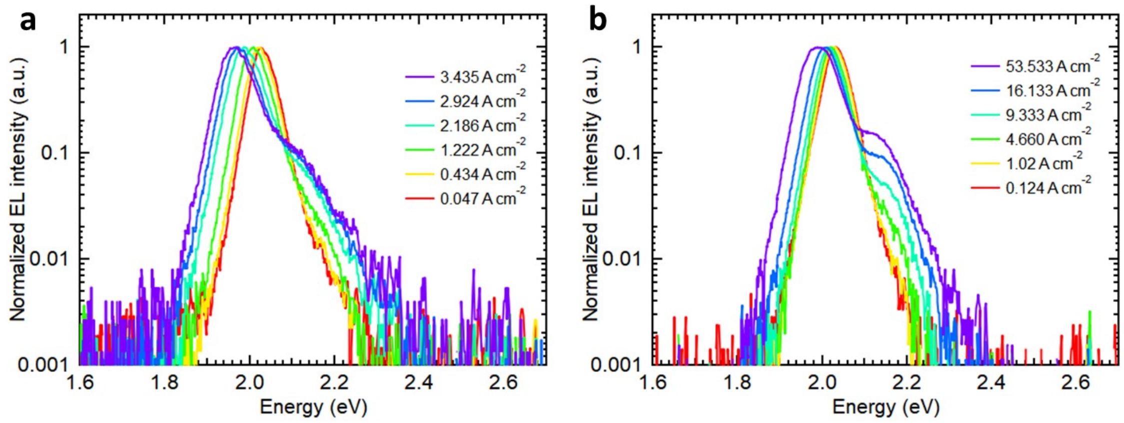

Supplementary Figure 6. EL spectra of cg-QDs as a function of current density (*j*). a, Normalized EL spectra of a planar LED driven by a d.c. bias as a function of *j*. **b,** Same for a current-focusing LED, also for a d.c. bias.

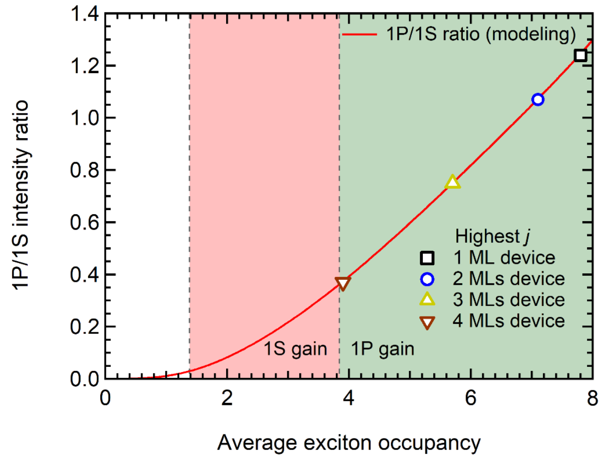

Supplementary Figure 7. The ratio of the 1P and the 1S EL band intensities as a function of average per-dot excitonic occupancy (⟨*N*⟩). The comparison of the calculated 1P/1S intensity ratio (Supplementary Note 2) with the measurements (symbols) is used to infer the average QD excitonic occupancy from the recorded EL spectra. The pink and green shadings show the ranges of 1S and 1P optical gain, respectively (Supplementary Note 3).

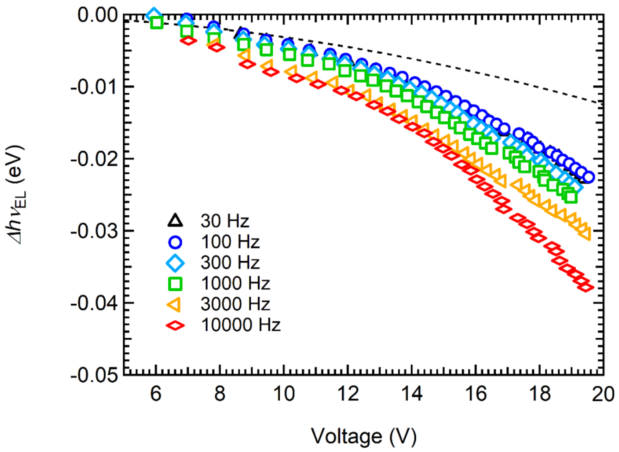

Supplementary Figure 8. The 1S EL peak shift ($\Delta h\nu_{EL}$) of the current focusing LED driven by short voltage pulses with the repetition rate (f_p) from 30 – 10,000 Hz. The 1S EL peak shift *versus* applied voltage (V) for pulse duration τ_p = 1 μs and f_p varied from 30 to 10,000 Hz (symbols). The dashed line is the projected contribution due to the Stark effect (Supplementary Fig. 3b). There is no significant difference in the EL peak shift for rates between 30 Hz and 100 Hz, which implies that the generated heat dissipates almost completely between the voltage pulses. For higher repetition rates (3,000 and 10,000 Hz), in addition to V, $\Delta h\nu_{EL}$ depends also on f_p, which indicates that the generated heat does not fully dissipate between the applied pulses.

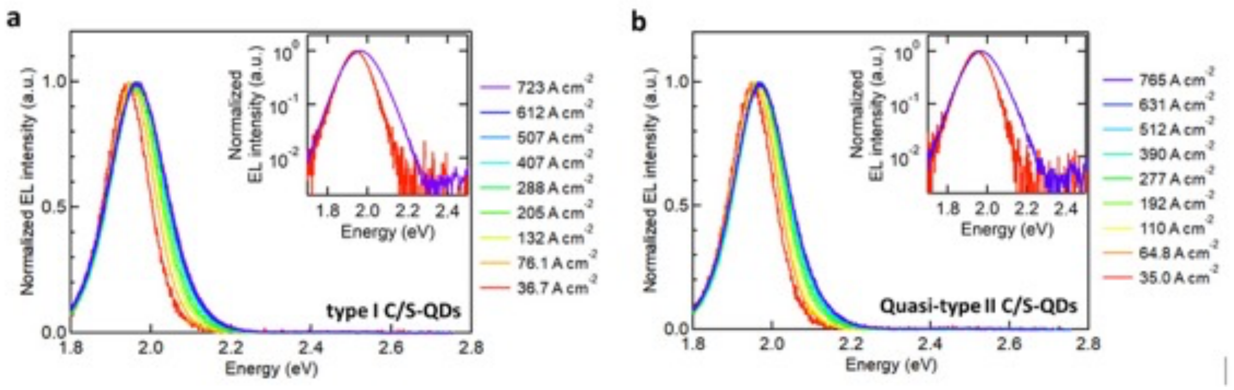

Supplementary Figure 9. EL spectra of traditional (nongraded) QDs incorporated into a current-focusing LED driven by a pulsed bias (τ_p = 1 μs). a, Normalized EL spectra of type-I nongraded CdSe/CdZnS for j up to 723 A cm^{-2}. **b,** Same for quasi-type-II nongraded CdSe/CdS QDs for j up to 765 A cm^{-2}. Despite extremely high current densities, neither of these samples shows a noticeable 1P EL, suggesting that the essential condition for realizing two-band (1S and 1P) EL is radial grading of the QD composition leading to strong suppression of Auger decay.

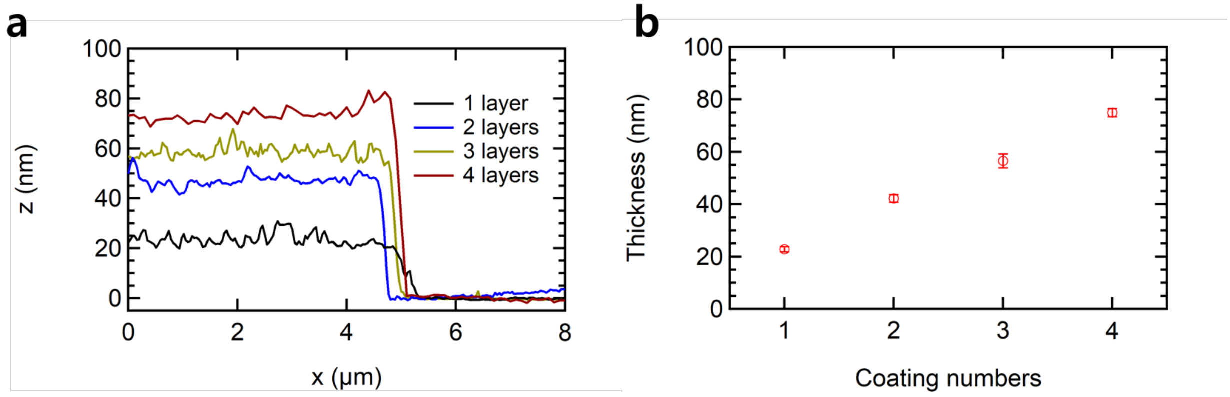

Supplementary Figure 10. Atomic-force-microscopy (AFM) measurements of the thickness of the cg-QD active layer. a, AFM scans across the edge of the cg-QD film indicate that its thickness increases in proportion with the number of the spin-coating cycles. **b,** The increments in the cg-QD film thickness are consistent with the QD size (~20 nm).

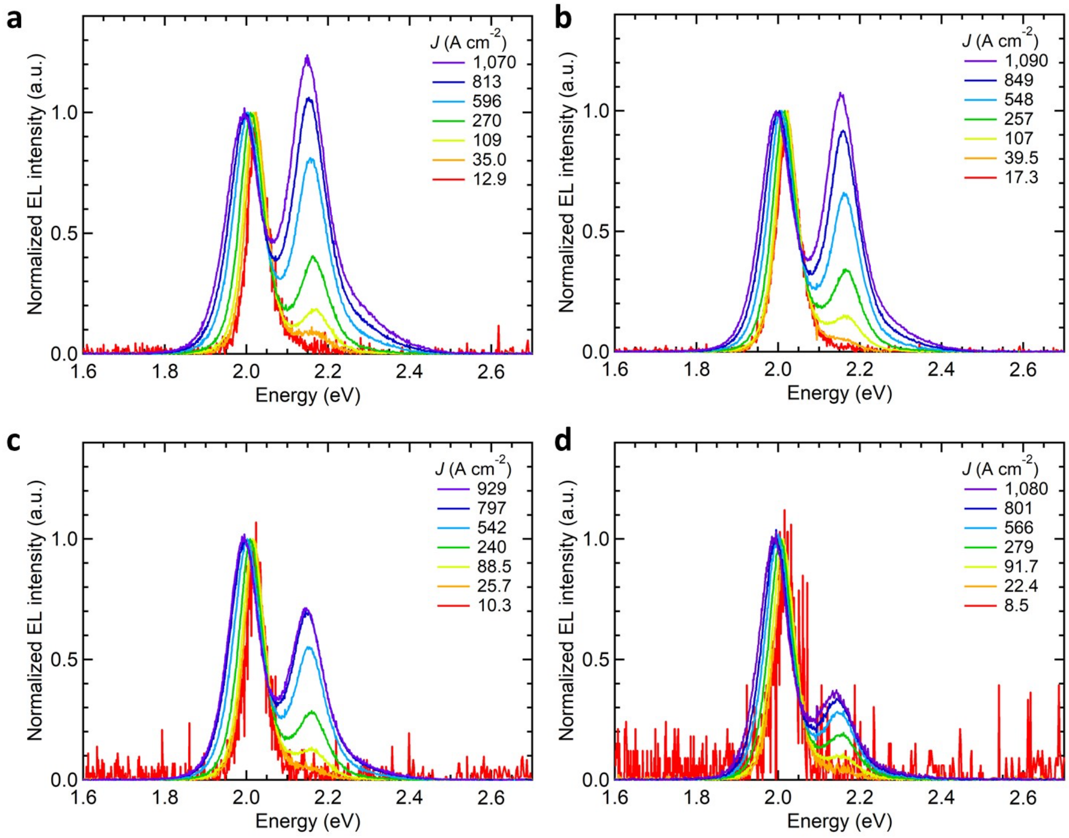

Supplementary Figure 11. EL spectra of LEDs made of a different number (1 to 4) of cg-QD layers. a, EL spectra of the LED made of the single cg-QD layer as a function of *j*. The device is driven by 1-μs pulses at a 100-Hz repetition rate. **b-d,** Same for devices containing 2 (**b**), 3 (**c**), and 4 (**d**) cg-QD layers.

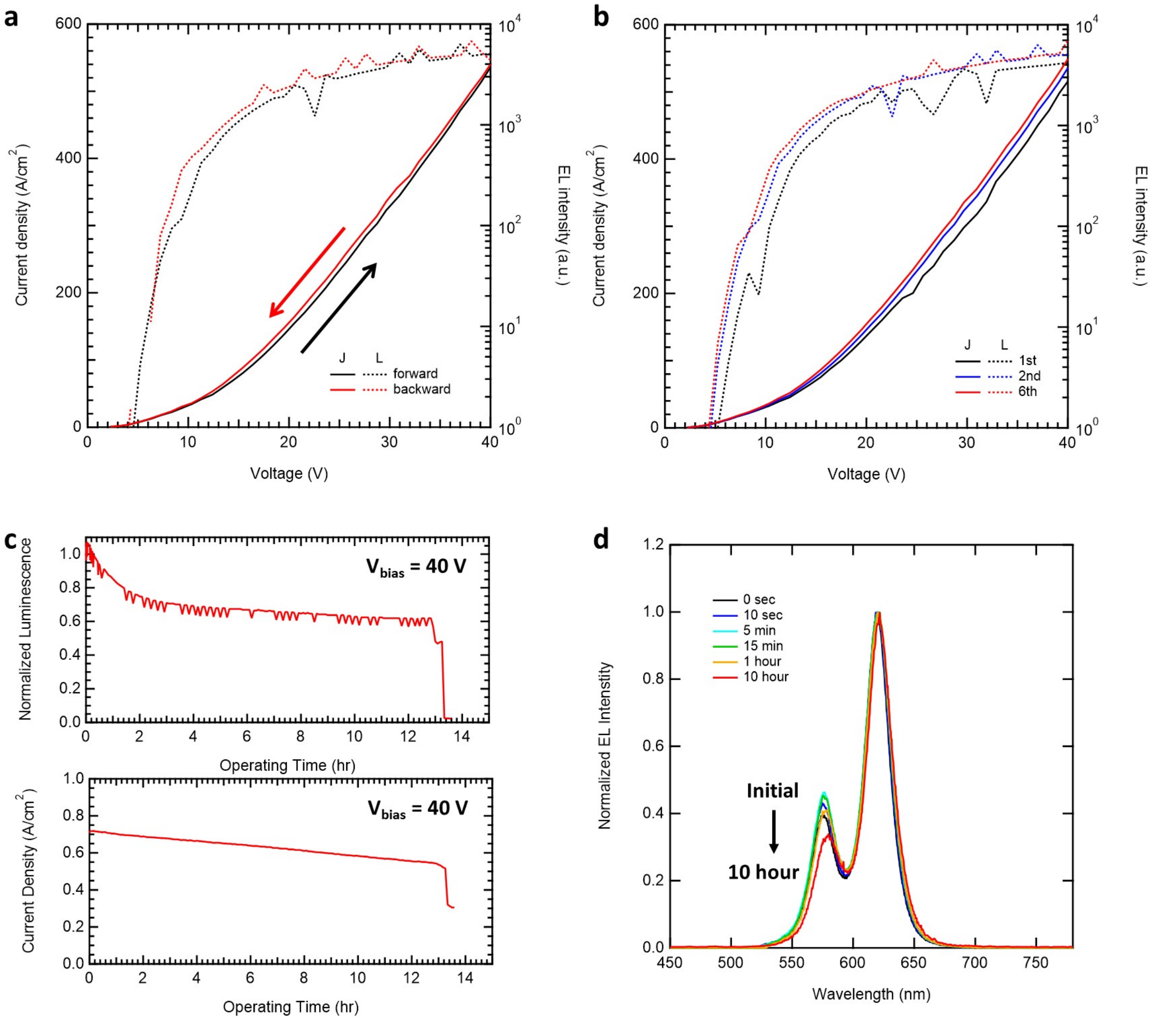

Supplementary Figure 12. Reproducibility and stability studies of short-pulse, current focusing LEDs. a, The $j-V_{total}$ characteristics do not show any hysteresis between the forward and the reverse scan. Here V_{total} is the total applied bias, which is a sum of the LED bias and the voltage drop across the connecting indium tin oxide (ITO) line (Supplementary Fig. 16). The hysteresis is also absent in the measurements of the EL intensity (L) versus V_{total}. **b,** Multiple scans show good reproducibility of the measured current density and EL intensity versus V_{total}. **c,** Current density (bottom) and L/L_0 (top; L_0 is the initial EL intensity) as a function of time of device operation (t). **d,** The EL spectra as a function of t. In all measurements, the LEDs were driven by 1-μs pulses at the 100 Hz repetition rate.

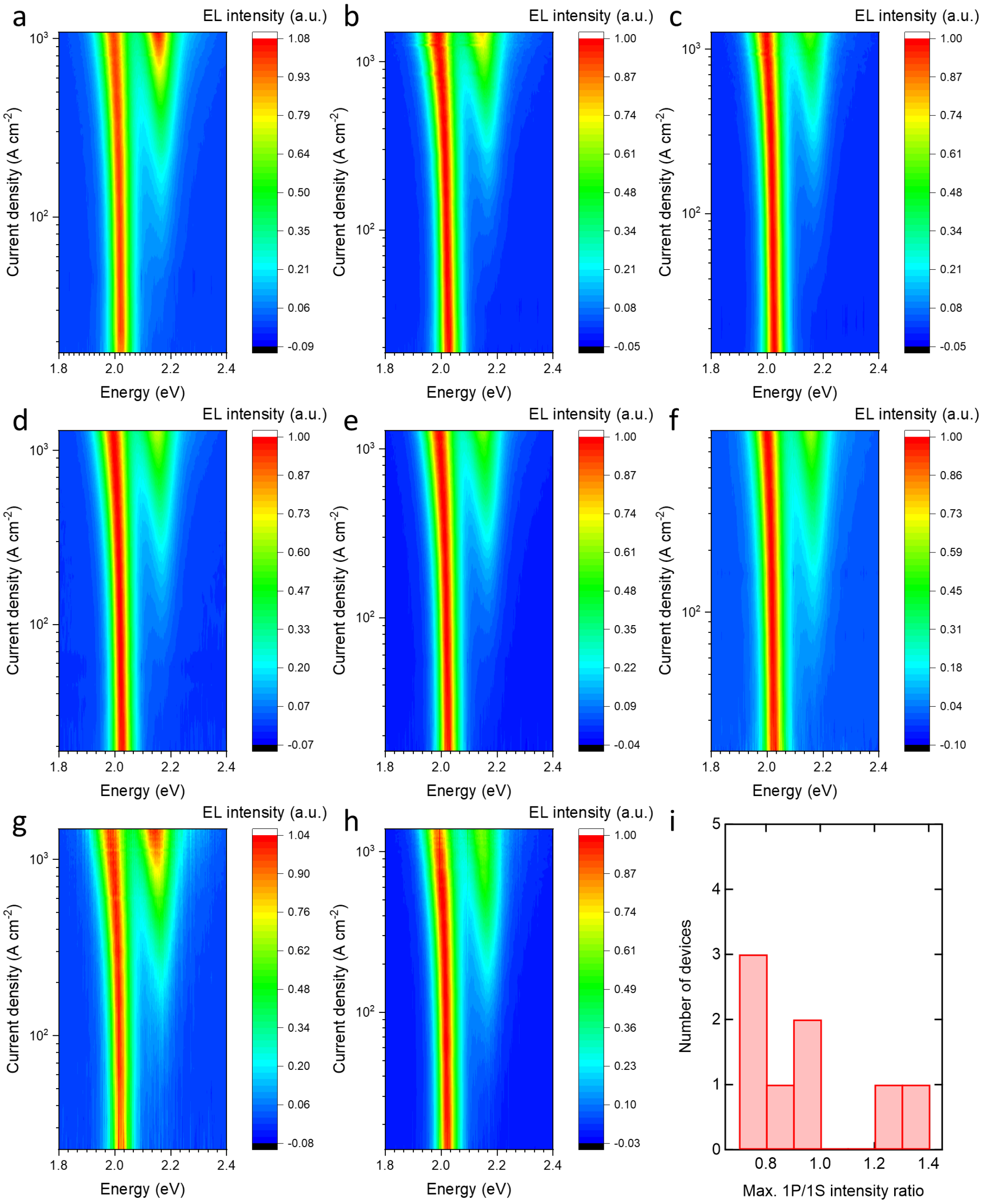

Supplementary Figure 13. Reproducibility tests of ultra-high-*j* LEDs. a-h, The EL spectra of 8 devices containing two cg-QD layers plotted as a function of *j*. The EL intensities are normalized at the position of the 1S emission peak. Seven devices out of the tested group showed j_{max} greater than 1 kA cm^{-2}. **i,** Histogram of the maximal 1P-to-1S intensity ratios realized for the tested devices. The observed ratio varied from 0.75 to 1.35 and on average was ~1. As discussed in the main article, this corresponds to the average per-dot excitonic occupancy of 6.8.

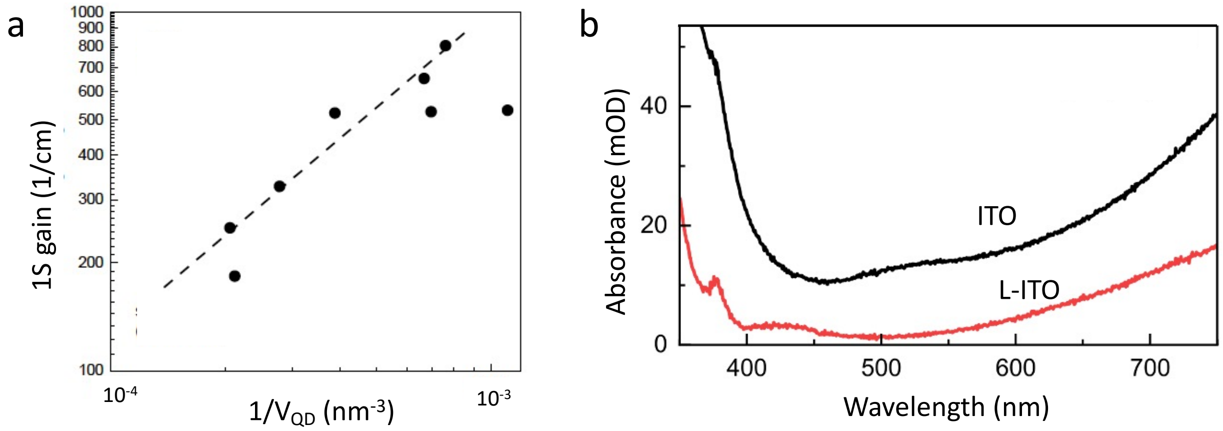

Supplementary Figure 14. a, 1S gain (G_{1S}) of cg-QDs as a function of total QD volume (V_{QD}) varied primarily through the thickness of the compositionally graded CdZnSe shell. The optical gain measurements were conducted in the regime of gain saturation using a variable stripe length technique applied to cg-QD films (~300 nm thickness) spin-coated onto glass slides. The measured values of $G_{1S,mat}$ scale approximately as inverse of the QD volume. This indicates that G_{1S} is directly proportional to the QD packing density. **b,** Absorbance spectra of films of standard indium tin oxide (ITO, black) and low-index ITO (L-ITO, red) for identical film thicknesses.

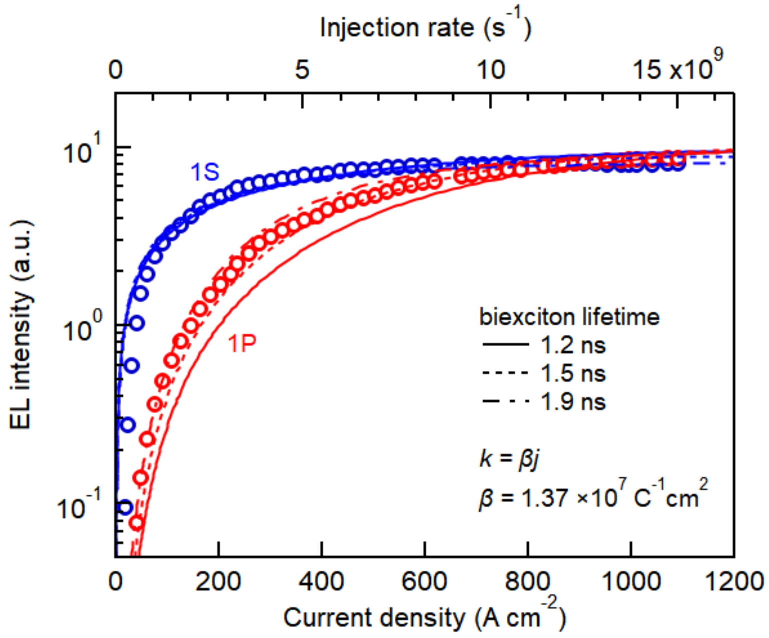

Supplementary Figure 15. Numerical calculations of the 1S and 1P EL intensities using the 'correlated injection' model. The measured 1S (blue circles) and 1P (red circles) EL intensities of the pulsed LED (same as in Fig. 3b of the main article) in comparison to calculations using the biexcition lifetime (τ_2) of 1.2 ns (solid line), 1.5 ns (dashed line) and 1.9 ns (dashed-dotted line) as a function of injection rate (g, top axis). Using β = 1.37×10^7 C^{-1} cm^2, we are able to match the calculations and the measurements. The corresponding current density obtained from $g = \sigma_e j/e$ is shown on the bottom axis (here e is the elementary charge and σ_e is the cg-QD electrical cross-section). The modeling conducted for τ_2 = 1.5 ns provides the best description of the measured 1S and 1P EL intensities. This value is close to that inferred from the measured PL dynamics (τ_2 = 1.2 ns; Supplementary Fig. 2b).

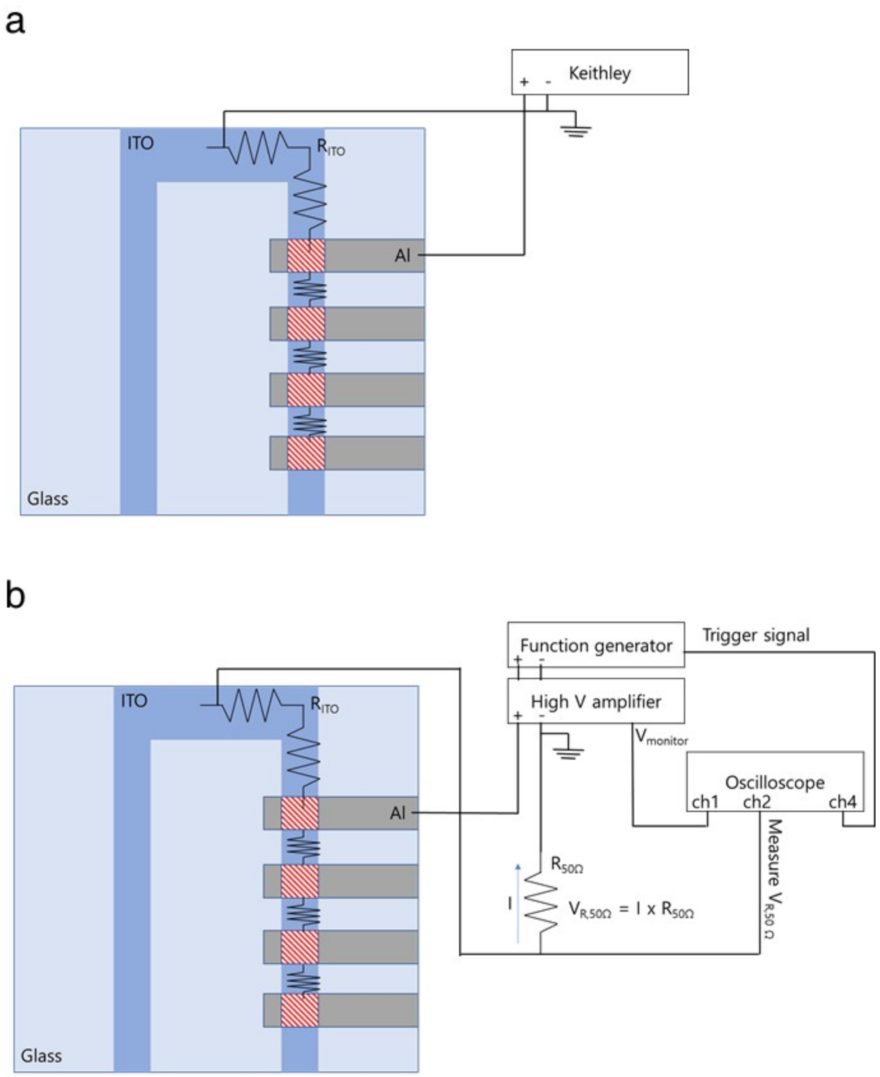

Supplementary Figure 16. Electrical schemes used in the *j*−*V* measurements. a, In the case of d.c. measurements, a Keithley source meter was used to bias a device and to measure its $j-V$ characteristics. **b,** In pulsed-excitation measurements, a high-voltage amplifier controlled by a function generator was used to generate a driving bias. An oscilloscope was used to read the voltage pulse. The current was inferred from a voltage drop across a 50 Ω resistor connected with the device in series. The cathode and anode probes were connected to the centers of the ITO and the Al pads, respectively. For both the d.c. and pulsed regimes, we obtained the voltage across the device by subtracting the voltage drops across the ITO line (from the cathode contact point to the device injection area) and the 50 Ω resistor from the applied voltage.

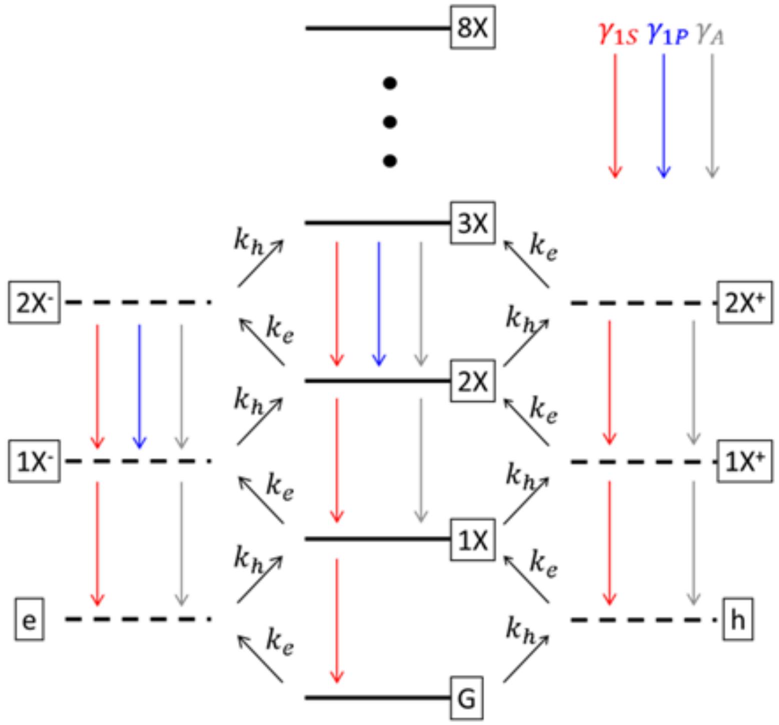

Supplementary Figure 17. A schematic illustration of the 'correlated injection' model. This model accounts for neutral (horizontal solid lines) and singly charged (horizontal dashed lines) excitonic states. The black arrows show the transitions between these states due to electron (rate k_e) and hole (rate k_h) injections. The grey and coloured arrows show recombination pathways due to Auger decay (grey arrow; rate γ_A) and radiative transitions involving the $1S_e$ (red arrow; rate γ_{1S}) and $1P_e$ (blue arrow; rate γ_{1P}) states.

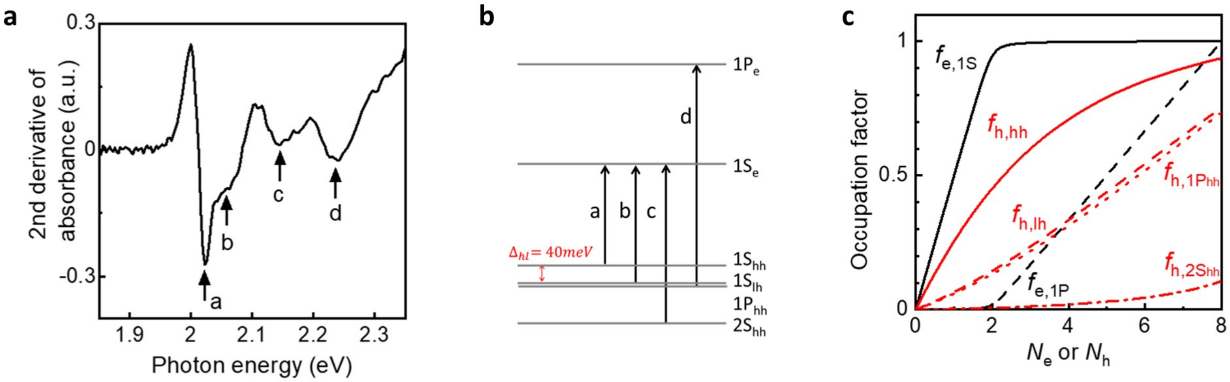

Supplementary Figure 18. The structure of optical transitions in cg-QDs. a, The positions of the four lowest optical transitions (2.023, 2.063, 2.145, and 2.238 eV labeled as 'a' through 'd', respectively) inferred from the 2nd-derivative of the linear absorption spectrum of the cg-QDs used in the present study. **b,** The features observed in **a** are assigned to the $1S_e$-$1S_{hh}$, $1S_e$-$1S_{lh}$, $1S_e$-$2S_{hh}$, and $1P_e$-$1P_{hh}$ transitions. **c,** Calculated occupation factors of the electron (black) and hole (red) states shown in **b** as a function of per-dot number of electrons (N_e) and holes (N_h).

Supplementary Table 1. The parameters used in the modeling of the EL spectra. Lifetimes, emission rates, and emission quantum yields of excitonic states with multiplicities (m) from 1 to 8. These include the total lifetime (τ), the radiative and Auger lifetimes (τ_r and τ_A, respectively), the 1S and 1P emission rates ($\gamma_{1S,r}$ and $\gamma_{1P,r}$, respectively), the total emission rate ($\gamma_r = \gamma_{1S,r} + \gamma_{1P,r}$), the 1S and 1P emission quantum yields (Q_{1S} and Q_{1P}, respectively), and the total emission quantum yield ($Q = Q_{1S} + Q_{1P}$). The emission rates are normalized to that of a single exciton ($\gamma_{1,r} = \gamma_{X,r} = 1/13.4$ ns^{-1}).

	1X	2X	3X	4X	5X	6X	7X	8X
τ (ns)	13.4*	1.2*	0.57	0.34	0.22	0.16	0.12	0.09
τ_r (ns)	13.4	3.69	2.11	1.42	1.04	0.79	0.63	0.52
τ_A (ns)		1.78	0.79	0.45	0.28	0.20	0.15	0.11
$\gamma_{1S,r}$	1	3.55	5.24	6.57	7.75	8.82	9.79	10.7
$\gamma_{1P,r}$	0	0	1.12	2.86	5.19	8.07	11.5	15.3
γ_r	1	3.55	6.36	9.43	12.9	16.9	21.3	26.0
Q_{1S}	1	0.32	0.22	0.17	0.13	0.11	0.088	0.072
Q_{1P}	0	0	0.05	0.07	0.08	0.09	0.10	0.10
Q	1	0.32	0.27	0.24	0.21	0.20	0.19	0.17

* Time constants obtained from the experiment (Supplementary Fig. 2).

Supplementary Table 2. Calculated mode confinement factors (*Γ*) for various layers of cg-QD LEDs studied in this work.

Layer (Thickness)	Γ (%) (λ= 620 nm)
Glass (Substrate)	10.1
ITO (150 nm)	56.1
ZnMgO (15 nm)	6.47
QD (80 nm)	23.2
TCTA (60 nm)	4.02
MoO_3 (10 nm)	0.08
Al (100 nm)	0.03
Sum	100

Supplementary Note 1. Modeling of device overheating

To treat the problem of heat accumulation in an operating light emitting diode (LED) based on colloidal quantum dots (QDs), we consider the interplay between heat generation due to a passing electric current (Joule heating) and heat dissipation due to thermal exchange with the environment. Since in the regime of high current densities ($j > 10\ A/cm^2$), a wall-plug efficiency ($P_{optical}/P_{electrical}$) of our devices is fairly low (<1%), we can assume that all electrical power, $P = jAV$ (here V is applied bias), is converted to heat[3]. Further, we neglect temperature gradients within an active device volume, which allows us to characterize it by a spatially uniform temperature T_d. To describe heat outflow, we will treat a surrounding environment is an ideal heat sink with temperature T_0, which in our case is room temperature, that is, $T_0 = 300$K.

In a steady state, heat generation is balanced by heat dissipation, that is,

$$AjV = K\Delta T, \tag{1}$$

where $\Delta T = T_d - T_0$, K is a heat exchange constant, and A is a charge injection area of the device. Based on Supplementary Equation (1), the device overheating in the steady-state regime can be computed from

$$\Delta T = AjVK^{-1}. \tag{2}$$

This expression suggests that the effect of overheating can be reduced by decreasing the injection area (that is, via 'current focusing') and/or boosting the heat exchange constant. The first of these measures reduces the overall amount of generated heat, and the second, enhances heat dissipation.

In the case of short-pulse excitation (pulse duration τ_p), the device temperature does not reach a steady state during an electrical pulse. In this situation, the evolution of device temperature can be described by

$$C\frac{d\Delta T}{d\mathrm{t}} = AjV - K\Delta T, \tag{3}$$

where C is a heat capacity of the active device volume. Based on Supplementary Equation (3), the time-dependent device temperature is given by

$$\Delta T(t) = \frac{AjV}{K}\left(1 - e^{-\frac{K}{C}t}\right). \tag{4}$$

Using Supplementary Equation (4), we obtain that the device temperature at the end of the pump pulse is

$$\Delta T(\tau_p) = \frac{AjV}{K}\left(1 - e^{-\frac{\tau_p}{\tau_T}}\right), \tag{5}$$

where $\tau_T = C/K$ is a characteristic heat dissipation time. In the long-pulse limit ($\tau_p \gg \tau_T$), Supplementary Equation (5) converges to that derived for the steady-state case (Supplementary Equation (2)). In the short-pulse limit ($\tau_p \ll \tau_T$), it yields

$$\Delta T(\tau_p) = \frac{AjV}{K}\frac{\tau_p}{\tau_T}. \tag{6}$$

This suggests that using short-pulse excitation, one can, in principle, reduce device overheating by a factor of τ_p/τ_T compared to steady-state excitation.

Supplementary Note 2. Modeling of two-band electroluminescence

To model j-dependent two-band electroluminescence (EL) spectra (see, e.g., Fig. 3a of the main article), we use a correlated injection model introduced in ref. [4]. A key premise of this model is that the electron and hole injections are mutually dependent. In particular, the presence of an electron in a QD facilitates the injection of a hole due to electron-hole attraction which lowers an injection barrier. Simultaneously, the Coulombic electron-electron repulsion impedes the injection

of an additional electron. As a result, QD excitation occurs preferentially as a sequence of correlated electron and hole injection steps. Based on this model, in our numerical calculations, we take into account only neutral and singly charged exciton and multiexciton states (Supplementary Fig. 17).

To calculate probabilities of neutral (P_m) and charged (P_m^+ and P_m^-) m-exciton states, we solve a system of coupled rate equations. For example, the temporal evolution of probability P_m (neutral m-exciton) is described by (Supplementary Fig. 17):

$$\frac{dP_m}{dt} = -\gamma_m P_m - (k_e + k_h)P_m + k_e P_{m-1}^+ + k_h P_{m-1}^- + \gamma_{m+1} P_{m+1}, \quad (7)$$

where γ_m is the m-exciton total decay rate, and k_e and k_h are the electron and hole injection rates, respectively. Similarly, the rate equations for positively and negatively charged m-excitons (γ_m^+ and γ_m^- decay rates, respectively) can be presented as

$$\frac{dP_m^+}{dt} = -\gamma_m^+ P_m^+ - k_e P_m^+ + k_h P_m + \gamma_{m+1}^+ P_{m+1}^+, \quad (8)$$

$$\frac{dP_m^-}{dt} = -\gamma_m^- P_m^- - k_h P_m^- + k_e P_m + \gamma_{m+1}^- P_{m+1}^-. \quad (9)$$

The total decay rates are computed as a sum radiative and nonradiative Auger rates (denoted by subscripts 'r' and 'A', respectively):

$$\gamma_m = \gamma_{m,r} + \gamma_{m,A}, \quad (10)$$

$$\gamma_m^+ = \gamma_{m,r}^+ + \gamma_{m,A}^+, \quad (11)$$

$$\gamma_m^- = \gamma_{m,r}^- + \gamma_{m,A}^-. \quad (12)$$

To calculate individual rates that appear in the right-hand side of Supplementary Equation (10 – 12), we characterize each state considered in the modeling in terms of the corresponding number of electrons (N_e) and holes (N_h). In particular, for a neutral m-exciton state $N_e = N_h = m$, while for the positively and negatively charged m-exciton, $N_e = N_h - 1 = m$ and $N_h = N_e - 1 = m$, respectively. Next, we apply a scaling law discussed, for example, in refs [4-6]. Based on the 'quadratic' scaling observed for CdSe QDs [6], the Auger rate of the (N_e, N_h) state can be presented as

$$\gamma_A(N_e, N_h) = \frac{\gamma_{2,A}}{4} N_e N_h \,, \tag{13}$$

where $\gamma_{2,A}$ is the biexciton Auger rate derived from the measurements. For our cg-QDs, $\gamma_{2,A} = 1.78$ ns^{-1} (derived from the overall biexciton lifetime of 1.2 ns and the radiative lifetime of 13.4 ns; Supplementary Fig. 2).

The radiative rate of the (N_e, N_h) state can be found via summation of rates of the participating dipole-allowed transitions. In our modeling, we consider the situation when the per-dot excitonic number does not exceed 8, which allows us to limit the summation to the $1S_e - 1S_{hh}$, $1S_e - 1S_{lh}$, and $1P_e - 1P_{hh}$ transitions (Fig. 2a, inset; main article). We further characterize the contributions from the S-type transitions by a single rate

$$\gamma_{1S,r}(N_e, N_h) = 2\gamma_{X,r} g_S f_{e,1S} \left(f_{h,1Sh} + f_{h,1Sl}\right), \tag{14}$$

and present the 1P-transtion radiative rate as

$$\gamma_{1P,r}(N_e, N_h) = 2\gamma_{X,r} g_P f_{e,1P} f_{h,1Ph} \,. \tag{15}$$

In the above two equations, $g_S = 2$ and $g_P = 6$ are the degeneracies of the S- and P-type states due to spin and azimuthal quantum number multiplicities and $\gamma_{X,r} = \gamma_{1,r}$ is the single-exciton radiative

rate, which is assumed to be 0.075 ns^{-1} based on the measured photoluminescence (PL) dynamics (Supplementary Fig. 2).

Quantities $f_{e,i}$ and $f_{h,j}$ are the occupation factors of individual quantized states with a certain combination of spin (up or down), principal (n = 1, 2, 3…), orbital angular momentum (L = 0, 1, 2, … for S, P, D…), and azimuthal (M = 0 for L = 0, M = -1, 0, +1 for L = 1, *etc.*) quantum numbers. These combinations are denoted by 'i' for electrons [*e.g.*, i = (up, 1, S_e, 0) for the spin-up 1S electron] and 'j' for holes [*e.g.*, j = (up, 1, S_{hh}, 0) for the spin-up 1S heavy hole]. The values of $f_{e,i}$ and $f_{h,j}$ are computed for given N_e and N_h from Supplementary Equations (21) and (22) (Supplementary Note 3) using a Fermi-Dirac distribution function which accounts for the thermal distribution of carriers across the QD quantized states.

After computing the radiative rates of individual transitions, we find the overall radiative rate $\gamma_r(N_e, N_h)$ from

$$\gamma_r(N_e, N_h) = \gamma_{1S,r}(N_e, N_h) + \gamma_{1P,r}(N_e, N_h). \quad (16)$$

Then, we obtain probabilities of various neutral and charged excitonic states from Supplementary Equations (7–9) and use them to calculate the intensities of the 1S and 1P EL features (I_{1S} and I_{1P}, respectively) from

$$I_{1S} = \sum_{m=1}^{7} \left[P_m \gamma_{1S,r}(m,m) + P_m^{+} \gamma_{1S,r}(m,m+1) + P_m^{-} \gamma_{1S,r}(m+1,m)\right] + P_8 \gamma_{1S,r}(8,8), \quad (17)$$

$$I_{1P} = P_2^{-} \gamma_{1P,r}(3,2) + \sum_{m=3}^{7} \left[P_m \gamma_{1P,r}(m,m) + P_m^{+} \gamma_{1P,r}(m,m+1) + P_m^{-} \gamma_{1P,r}(m+1,m)\right] + P_8 \gamma_{1P,r}(8,8), \quad (18)$$

where $\gamma_{1S,r}(N_e, N_h)$ and $\gamma_{1P,r}(N_e, N_h)$ are computed using Supplementary Equation (14) and (15), respectively.

To relate these calculations to the EL measurements, we follow ref. [4] and assume that the hole and electron injection rates are equal to each other and, further, are proportional to the current density (j) passing through the device, that is,

$$k_e = k_h = k = \beta j, \tag{19}$$

where β is a proportionality constant.

Here, we apply the above formalism to model the j-dependent 1S and 1P EL intensities (Fig. 3b of the main article and Supplementary Fig. 15). In our modeling, the emission rates are calculated from Supplementary Equations (14–16) based on the measured single-exciton radiative rate (Supplementary Table 1), and β and τ_2 are used as adjustable parameters. We observe the best correspondence between the experiment and the modeling for β = 1.37×10^7 C^{-1} cm^2 and τ_2 = 1.5 ns (Supplementary Fig. 15). Importantly, the biexciton lifetime obtained from the calculations is close to that inferred from the measured PL dynamics ($\tau_2 = \tau_{XX}$ = 1.2 ns; Supplementary Fig. 2b).

Supplementary Note 3. Modeling of optical gain thresholds

The gain threshold for the transition, which couples the i-electron and the j-hole states, can be found from condition

$$f_{e,i} + f_{h,j} = 1, \tag{20}$$

there $f_{e,i}$ and $f_{h,j}$ are state's occupation factors calculated according to the Fermi-Dirac distribution function:

$$f_{e,i} = \frac{1}{1 + e^{\frac{E_{e,i} - \mu_e}{kT}}} \text{ and } f_{h,j} = \frac{1}{1 + e^{\frac{E_{e,j} - \mu_h}{kT}}}, \tag{21}$$

where T is the temperature, $E_{e,i}$ and $E_{h,j}$ are the electron and hole state energies, and μ_e and μ_h are the corresponding chemical potentials. In the case when a QD is occupied with N_e electrons and N_h holes, μ_e and μ_h can be found from:

$$N_e = \sum_i g_{e,i} f_{e,i} \text{ and } N_h = \sum_j g_{h,j} f_{h,j}, \qquad (22)$$

where the summation extends over states with different principle and orbital angular momentum numbers (*e.g.*, $1S_e$, $1S_{hh}$, $1S_{lh}$, *etc.*). The summation over states with different azimuthal and spin quantum numbers is accounted for by the degeneracy factors $g_{e,i}$ and $g_{h,j}$ that are equal to 2 and 6 for the S- and P-type states, respectively.

Applying this approach to cg-QDs with the electronic states depicted in Supplementary Fig. 18, we obtain the following room temperature gain thresholds expressed in terms of the per-dot average excitonic occupancy: $\langle N \rangle_{th,gain}$ = 1.35 ($1S_e - 1S_{hh}$), 1.79 ($1S_e - 1S_{lh}$), 3.84 ($1P_e - 1S_{hh}$), 5.23 ($1P_e - 1S_{lh}$), 5.32 ($1P_e - 1P_{hh}$). In addition to the allowed transitions ($1S_e - 1S_{hh}$, $1S_e - 1S_{lh}$, and $1P_e - 1P_{hh}$), in these calculations, we have also considered nominally forbidden $1P_e - 1S_{hh}$ and $1P_e - 1S_{lh}$ transitions. The population inversion of the $1P_e - 1S_{hh}$ occurs before that of the $1P_e - 1P_{hh}$ transition, and, as was observed in ref. [4], it determines the optical gain threshold for the cg-QDs. Due to optical selection rules, the $1P_e - 1S_{hh}$ transition is in principle forbidden. However, as was invoked previously, this transition can become weakly allowed due to parity violation caused, for example, by the electric field associated with multiexciton states, leading to mixing of the S and P states[4,7].

Supplementary References

1 Empedocles, S. A. & Bawendi, M. G. Quantum-confined Stark effect in single CdSe nanocrystallite quantum dots. *Science* **278**, 2114-2117 (1997).

2 Park, K., Deutsch, Z., Li, J. J., Oron, D. & Weiss, S. Single molecule quantum-confined stark effect measurements of semiconductor nanoparticles at room temperature. *ACS Nano* **6**, 10013-10023 (2012).

3 Yoshida, K., Nakanotani, H. & Adachi, C. Effect of Joule heating on transient current and electroluminescence in *p-i-n* organic light-emitting diodes under pulsed voltage operation. *Org. Electron*. **31**, 287-294 (2016).

4 Lim, J., Park, Y.-S. & Klimov, V. I. Optical gain in colloidal quantum dots achieved with direct-current electrical pumping. *Nat. Mater.* **17**, 42-49 (2018).

5 Klimov, V. I. Multicarrier interactions in semiconductor nanocrystals in relation to the phenomena of auger recombination and carrier multiplication. *Annu. Rev. Condens. Matter Phys.* **5**, 285-316 (2014).

6 Klimov, V. I., McGuire, J. A., Schaller, R.D. & Rupasov, V. I. Scaling of multiexciton lifetimes in semiconductor nanocrystals. *Phys. Rev. B* **77**, 195324 (2008).

7 Caruge, J.-M., Chan, Y., Sundar, V., Eisler, H. J. & Bawendi, M. G. Transient photoluminescence and simultaneous amplified spontaneous emission from multiexciton states in CdSe quantum dots. *Phys. Rev. B* **70**, 085316 (2004).
